# Comparative analysis of chloroplast genomes and phylogenetic relationships in the endemic Chinese bamboo *Gelidocalamus* (Bambusoideae)

**DOI:** 10.3389/fpls.2024.1470311

**Published:** 2024-11-11

**Authors:** Chengkun Wang, Yonglong Li, Guangyao Yang, Wengen Zhang, Chunce Guo

**Affiliations:** Jiangxi Provincial Key Laboratory of Improved Variety Breeding and Efficient Utilization of Native Tree Species, Forestry College, Jiangxi Agricultural University, Nanchang, China

**Keywords:** *Gelidocalamus* Wen, phylogenetic relationships, plastome evolution, molecular markers, divergence times

## Abstract

**Introduction:**

*Gelidocalamus* Wen is a small yet taxonomically challenging genus within the Arundinarieae tribe. Recent molecular studies have suggested it may not be monophyletic. However, limited species sampling and insufficient molecular marker information have resulted in poorly resolved phylogenetic relationships within this genus.

**Methods:**

The complete chloroplast genomes covering all 16 species and one variant of *Gelidocalamus* were sequenced, and comparative analyses were conducted. Phylogenetic analyses were performed using different molecular markers, including chloroplast data, the nuclear ribosomal DNA (nrDNA) repeats region, and 29 mitochondrial protein-coding genes. Additionally, the divergence times of *Gelidocalamus* were estimated to reveal their evolutionary history.

**Results:**

The plastomes of *Gelidocalamus* ranged in size from 139,500 bp to 139,801 bp, with a total of 137 identified genes, including 90 protein-coding genes, 39 tRNA genes, and 8 rRNA genes. The size of the nrDNA repeats ranged from 5,802 bp to 5,804 bp. Phylogenetic analysis based on chloroplast data revealed that *Gelidocalamus* is polyphyletic, with different subclades distributed within the IV and V clades. However, phylogenetic analysis based on nrDNA and mitochondrial genes did not effectively resolve the relationships within the genus.

**Discussion:**

Comparative analysis of chloroplast genomes indicated that *Gelidocalamus* shares a high degree of similarity with closely related genera in terms of chloroplast genome collinearity, codon usage bias, and repetitive sequences. Divergence time estimation suggests that it is a relatively young group, with all members appearing successively over the past four million years. The complex phylogenetic patterns may arise from the rapid radiation of Arundinarieae. This study provides a preliminary foundation for further in-depth research on the phylogeny, genomic structural features, and divergence times of this genus.

## 1 Introduction

*Gelidocalamus* Wen, a small genus belonging to the Arundinarieae tribe, is endemic bamboo to the humid evergreen broad-leaved forests of eastern China. Established in 1982, the genus comprises approximately 16 species and one variety (Wen, 1982; Li et al., 2006; Zhang et al., 2017; Cai et al., 2021; Wang et al., 2023; Li et al., 2023). *Gelidocalamus* is characterized by several distinct morphological features, such as multiple branches per node, cylindrical and grooveless internodes, persistent sheaths, a single foliage leaf on each ultimate branch (except *G. multifolius* B. M. Yang) (Yang, 1986), and semelauctant inflorescence. One notable characteristic is the emergence of new shoots during the autumn and winter (Liu et al., 2017; Nie et al., 2018). This genus primarily inhabits hilly and low-mountain areas south of the Yangtze River, at elevations below 1,000 m (Li et al., 2016; Nie et al., 2018). *Gelidocalamus* typically grows alongside mountain streams, gullies, and waterways under the canopy of evergreen broad-leaved forest, forming distinctive strip-like patterns. As a stable component of middle-subtropical forests, the *Gelidocalamus* plays a crucial role in water conservation and ecological balance. Additionally, it is valued for its elegant foliage and edible young shoots, offering significant potential for ornamental and culinary uses. However, as a complex genus, some bamboo species are scarce, and our current understanding is primarily based on morphological studies. Little is known about their phylogenetic relationships, origin, and evolution, which restricts further utilization and conservation efforts (Wen, 1982; Liao, 1991; Zhang et al., 2017).

Traditionally, bamboo species classification has relied on vegetative organ characteristics due to the unpredictability of bamboo flowering phenology. However, bamboo species exhibit a high degree of homogeneity in vegetative traits, posing significant challenges to taxonomy and systematics (Peng et al., 2008; Zeng et al., 2010). Molecular phylogenetic studies have sought to address these challenges using various markers such as the internal transcribed spacer (ITS) and single-copy nuclear genes like *Granule-Bound Starch Synthase I* (*GBSSI*) gene. Despite these efforts, the resolution of phylogenetic relationships based on ITS and single-copy genes has been limited (Peng et al., 2008; Zhang et al., 2012; Tong et al., 2020). In recent years, the rapid advancement of Restriction-site Associated DNA sequencing (RAD) sequencing technology has significantly enhanced our understanding of the phylogeny and evolutionary diversification of bamboo species. Ye et al. (2019) employed ddRAD-seq data to reconstruct the phylogeny of bamboo species, focusing on 178 individuals representing alpine bamboo. Similarly, Guo et al. (2021) utilized ddRAD-seq data to establish a phylogenetic framework for temperate bamboo involving 213 individuals from 200 species across 32 genera. These studies provided high-resolution phylogenies for temperate bamboo species and offered insights into their diversification patterns. Additionally, the investigation into the evolution of key traits sheds light on the complex evolutionary history of temperate bamboo (Ye et al., 2019; Guo et al., 2021). In addition, the use of chloroplast gene fragments or complete genomes has provided superior resolution (Triplett and Clark, 2010; Zeng et al., 2010; Yang et al., 2013; Attigala et al., 2014; Zhou et al., 2022). Since the sequencing of the complete chloroplast genomes of *Nicotiana tabacum* and *Marchantia polymorpha* in 1986 (Ohyama et al., 1986; Sugiura et al., 1986), a wealth of chloroplast genome data have become available. Chloroplast genomes are favored for phylogenetic studies due to their highly conserved gene structures, moderate evolutionary rates, uniparental inheritance, minimal influence from paralogs, and ease of genome extraction (Zhang and Li, 2011; Wang et al., 2012; Li et al., 2020). Using plastid loci or chloroplast genome data, the phylogenetic framework of the Arundinarieae tribe has been progressively constructed and refined (Triplett and Clark, 2010; Zeng et al., 2010; Yang et al., 2013; Attigala et al., 2014; Ma et al., 2014; Zhang et al., 2018, Zhang et al., 2020). This tribe is robustly supported as a monophyletic group, with a sister relationship between the Bambuseae and Olyreae tribes. In contrast, the phylogenetic relationships constructed from chloroplast genes differ from those based on nuclear genes. In the nuclear gene tree, Olyreae diverges first, with Arundinarieae and Bambuseae forming sister groups. In the chloroplast tree, however, Arundinarieae diverges first, and Bambuseae clusters with Olyreae (Guo et al., 2021; Gallaher et al., 2022; Huang et al., 2022).

Although a clear framework has been defined in the Arundinarieae tribe. However, studies on the chloroplasts of *Gelidocalamus* have been limited, and only a few species have been sporadically sampled. The relationships within *Gelidocalamus* and its associations with closely related genera such as *Indocalamus* Nakai, *Ferrocalamus* Hsueh & Keng f., *Shibataea* Makino ex Nakai, *Sinosasa* L. C. Chia ex N. H. Xia, Q. M. Qin & Y. H. Tong, and others within clades IV and V remain unclear (Peng et al., 2008; Triplett and Clark, 2010; Zeng et al., 2010; Zhang et al., 2019; Qin et al., 2020). According to the chloroplast genome-based study, *G. stellatus*, *G. latifolius*, and *G. solidus* are situated within clade V. *G. stellatus* and *G. latifolius* form into a clade with *Indocalamus*, while *G. solidus* clustered separately with *Chimonobambusa ningnanica*. Additionally, *G. tessellatus* forms a clade with the genus *Shibataea* (Guo et al., 2021). Interestingly, the phylogenetic relationships within the Arundinarieae tribe, as inferred from ddRAD data, included a total of 13 *Gelidocalamus* species. Six of these species (*G. annulatus*, *G. tessellatus*, *G. latifolius*, *G. stellatus*, *G. multifolius*, and *G. monophyllus*) clustered into a monophyletic branch. Three species (*G. solidus*, *G. subsolidus*, and *G. albopubescens*) formed a monophyletic lineage that was sister to *Indocalamus* and *Bashannia*, but their phylogenetic relationships conflicted dramatically with those based on chloroplast genome data. The remaining four (*G. dongdingensis*, *G. rutilans*, *G. kunishii*, *G. longiinternodus*) were nested within *Indocalamus*, which significantly conflicted with chloroplast genome results. Additionally, the clades IV and V, where *Gelidocalamus* is situated, were reconfigured to form a new *Indocalamus* + *Gelidocalamus* + *Chimonocalamus* branch (Guo et al., 2021). This article argues that such a significant nucleoplasmic conflict reflects the differing evolutionary histories of the nucleoplasm.

In this study, we collected 27 samples representing 16 species and one variant of *Gelidocalamus*, along with plastomes from closely related genera obtained from the NCBI database and the data matrix of Guo et al. (2021). We re-annotated and examined these plastomes, assembling and annotating newly sequenced plastomes to investigate the phylogenetic relationships within the *Gelidocalamus*. We also assembled nrDNA repeats and mitochondrial coding genes from low-depth data to perform phylogenetic inference and estimate divergence times for *Gelidocalamus*. Our objectives are to (1) explore the phylogenetic relationships among *Gelidocalamus* species while assessing the contributions of molecular markers, (2) understand the structural characteristics and sequence variations of the chloroplast genomes of *Gelidocalamus* and its closely related species, and (3) reveal the evolutionary history of *Gelidocalamus*.

## 2 Materials and methods

### 2.1 Sampling, DNA extraction, sequencing, assembly, and annotation

To conduct a comprehensive study on the relationships of *Gelidocalamus*, we performed field surveys at both the type and nontype localities of each species. Mature leaves were collected from individuals at these localities, and all voucher specimens were deposited in the herbarium of Jiangxi Agricultural University, China (JXAU).

Genomic DNA was isolated from dried foliage leaves over silica gel using a modified CTAB method (Murray and Thompson, 1980). Illumina paired-end libraries (2 × 150 bp) were constructed and sequenced by Novogene Bioinformatics Technology Co. Ltd. (Beijing, China), yielding approximately 8 GB of raw data per sample. To enhance assembly accuracy, FastQC 0.11.9 (https://www.bioinformatics.babraham.ac.uk/projects/fastqc) and Fastp 0.12.4 (Chen et al., 2018) were used with default parameters to filter out unpaired and low-depth reads.

In the assembly process, the chloroplast genome was assembled using GetOrganelle 1.7.4 (Jin et al., 2020) with k-mers of 45, 65, 85, 105, and 121. Following this, the filtered reads underwent processing through Bandage (Wick et al., 2015) to facilitate the attachment of chloroplast genome scaffolds. The clean reads were mapped to the draft genome using Geneious 9.1.4 (Kearse et al., 2012) to check the concatenation of contigs. The resultant chloroplast genome sequences were annotated using CPGAVAS2 (Shi et al., 2019), followed by a meticulous manual review in Geneious. To visualize the sequenced chloroplast genomes, Chloroplot (Zheng et al., 2020) was employed. Similarly, the assembly of nrDNA repeats was executed using GetOrganelle with k-mers of 35, 85, and 115, supplemented by manual corrections within Geneious. For the mitochondrial genome assembly, Geneminer (Xie et al., 2023) was utilized under default parameters, referencing the complete mitochondrial genomes of *Bambusa oldhamii* (EU365401) and *Ferrocalamus strictus* (JN120789). The resulting output underwent further enhancement through subsequent corrections in Geneious.

### 2.2 Phylogenetic analyses

To elucidate the phylogenetic relationships among 16 species and one variant within *Gelidocalamus*, as well as their relationships with related genera, we selected and reannotated additional 44 plastomes from 24 genera (**Supplementary Table S1**) to represent the 12 major clades of the Arundinarieae tribe. Additionally, we chose four plastomes (*Dendrocalamus latiflorus*, *Bambusa multiplex*, *Olyra latifolia*, and *Raddia brasiliensis*) from the Bambuseae and Olyreae tribes as outgroups. All plastomes were sourced from the NCBI database (https://www.ncbi.nlm.nih.gov/) and the data matrix of Guo et al. (2021).

Phylogenetic relationships were inferred using both maximum likelihood (ML) and Bayesian inference (BI) methods. For the ML analysis, whole chloroplast genomes and one IR region sequence were aligned using MAFFT 7.450 (Katoh and Standley, 2013) and trimmed with trimAl (Capella-Gutiérrez et al., 2009), respectively. IQ-TREE (Nguyen et al., 2015) was employed with 5,000 bootstrap replications, and the best-fit BIC model GTR + F + I + G4 was selected using ModelFinder (Kalyaanamoorthy et al., 2017). For Bayesian Inference (BI), MrBayes 3.2.6 (Ronquist et al., 2012) was utilized with the same model. Markov Chain Monte Carlo (MCMC) simulations ran for 20,000,000 generations, ensuring an average standard deviation of split frequencies (ASDFs) < 0.01, with sampling every 1,000 generations. The initial 25% burn-in samples were discarded, and the optimized topology was obtained. Nuclear ribosomal DNA (nrDNA) sequences and mitochondrial coding genes were also used in the phylogenetic analysis, using the same methods as described above.

### 2.3 Estimation of divergence time

To elucidate the origin and evolutionary history of the *Gelidocalamus*, divergence times were estimated from chloroplast genomic data using the BEAST v2.6.3 (Bouckaert et al., 2019). Estimating divergence times within the Arundinarieae tribe or even the Bambusoideae is challenging due to the scarcity of credible fossil evidence and the ambiguity and fragmentary nature of several bamboo fossil records (Guo et al., 2021). As a result, we adopted a conservative approach by utilizing a Bambusoideae fossil, Bambusoideae cf. *Chusquea* (Strömberg, 2011), to calibrate the crown Bambusoideae, and supplemented this with three additional secondary calibration points to assist in estimating divergence times (Zhang et al., 2016). Calibration point information is as follows: (1) Bambusoideae cf. *Chusquea* (35–90 Mya); (2) Arundinarieae crown (6.88–20.96 Mya, median age: 12.72 Mya); (3) clade IV crown (2.166.58 Mya, median age: 4.01 Mya); and (4) Clade V crown (1.24–3.82 Mya, median age: 2.38 Mya). Each calibration point was implemented as a uniform distribution between the minimal and maximal age of the constraint. We used a Yule process tree prior, the uncorrelated lognormal relaxed clock model, and the HKY substitution model. The MCMC chain length was set at 250,000,000, with a sampling frequency of 1,000. To assess parameter convergence, we utilized Tracer v1.7.2, stipulating an effective sample size (ESS) exceeding 200. Subsequently, we summarized the age statistics for internal nodes using TreeAnnotator v2.6.6.

### 2.4 Plastome structure comparison and sequence divergence analyses

To explore the structural variations in chloroplast genomes within *Gelidocalamus*, we statistically analyzed the total length of the chloroplast genome, the number of genes, and the GC content of each region using Geneious (Kearse et al., 2012). Subsequently, all sequences were aligned and compared using MAFFT (Katoh and Standley, 2013). The alignments were then analyzed for potential rearrangements and inversions through covariance analysis using the mauve plugin within Geneious. Furthermore, events of expansion and contraction within the inverted repeat (IR) regions among the chloroplast genomes were visualized using the IRscope online program (Amiryousefi et al., 2018). In order to further understand the sequence divergence and hypervariable regions detected, the nucleotide diversity (pi) values of plastomes were calculated using DnaSP v.5.1 (Librado and Rozas, 2009) with a sliding window analysis.

### 2.5 Codon usage analyses

To determine the codon usage pattern of *Gelidocalamus*, we extracted protein-coding genes from chloroplast genomes using PhyloSuite (Zhang et al., 2019). Subsequently, we calculated the relative synonymous codon usage (RSCU) values for each protein-coding gene using CodonW (https://codonw.sourceforge.net/) and visualized the resulting data using TBtools (Chen et al., 2020). An RSCU value equal to 1.00 indicates no codon usage bias, an RSCU value less than 1.00 suggests less frequent codon usage, while an RSCU value greater than 1.00 indicates the presence of codon usage bias.

### 2.6 Repeats and SSR analyses

To detect repetitive sequences in chloroplast genome sequences, an online tool MiSa-web (Beier et al., 2017) was employed for the analysis of each genome. The parameter settings included a minimum repeat count of 10 for mononucleotide repeats, a minimum repeat count of five for dinucleotide repeats, a minimum repeat count of four for trinucleotide repeats, and a minimum repeat count of three for tetranucleotide to hexanucleotide repeats. Simultaneously, the identification of scattered repeat sequences within the chloroplast genomes was conducted using REPuter (Kurtz et al., 2001). This analysis covered forward, reverse, complementary, and palindromic repeat sequences, with parameter settings specifying a Hamming Distance of 3, a Minimal Repeat Size of 30, and a Maximum Computed Repeats of 80.

## 3 Results

### 3.1 Sampling, sequencing, and data assembly

In this study, fresh leaves of *Gelidocalamus* were collected for molecular analyses. A total of 27 populations were recorded, comprising 15 type localities representing 14 species and one variety (**Supplementary Table S1**). The morphological diversity of *Gelidocalamus* was documented and photographed (**Supplementary Figure S1**), and specimens were collected during the field survey. However, surveys for two species, *G. kunishii* and *G. fengkaiensis* (recently published), were not conducted. Illumina sequencing yielded a range of 39,708,004 to 207,484,842 paired-end clean reads across 27 *Gelidocalamus* samples (**Supplementary Table S2**). The complete chloroplast genome, nrDNA repeat segments (including the 18S, ITS1, 5.8S, ITS2, and 26S regions), and 29 shared mitochondrial genes were obtained from each species (**Supplementary Figure S2**).

**Figure 1:**
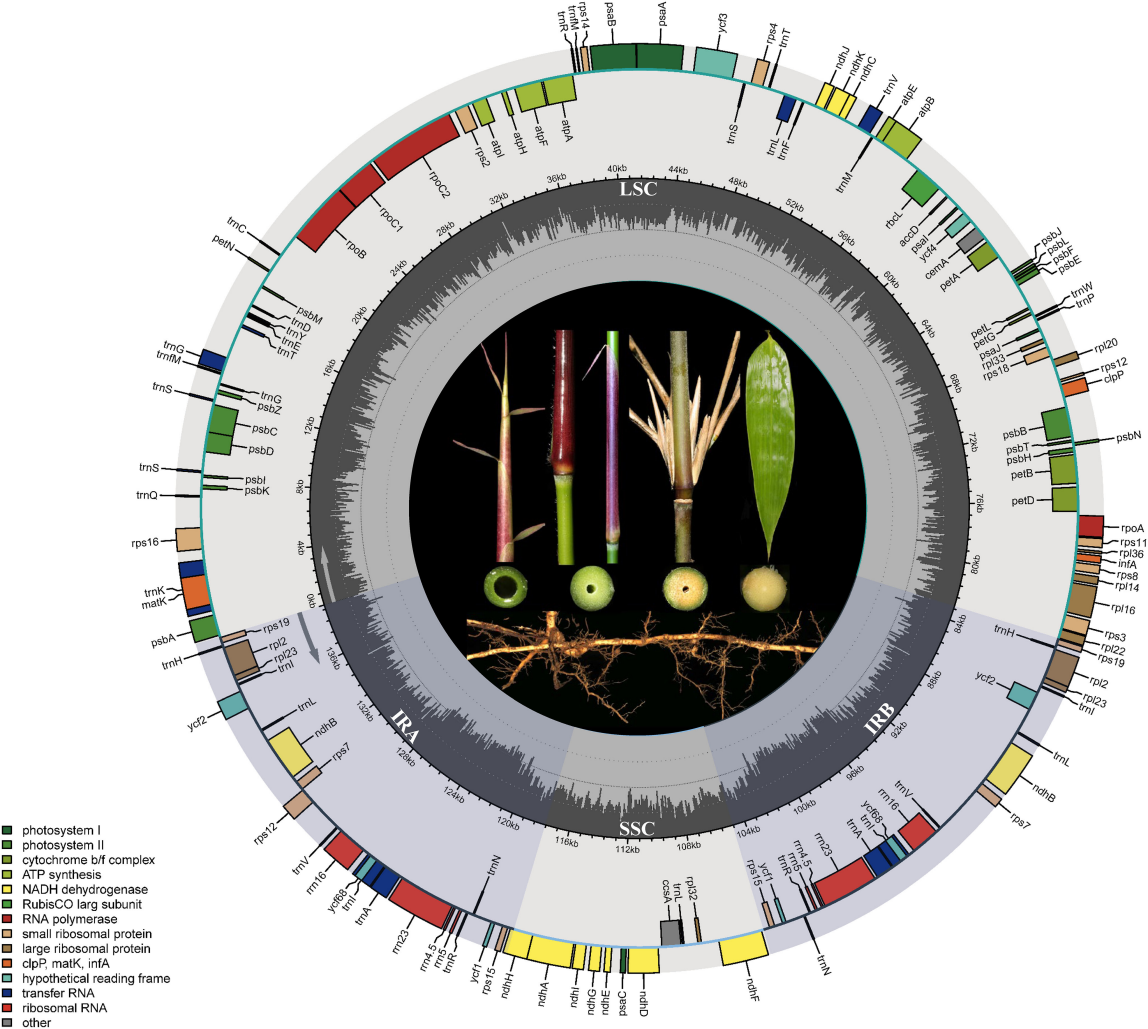
Gene maps of the chloroplast genome in *Gelidocalamus*. The whole genome length is 139,500–139,801 bp. Colored bars indicate different functional gene groups. The sector shading represents the inverted repeat regions (IRa and IRb), which divide the genome into small (SSC) and large (LSC) single-copy regions. The dark gray inner circle shows GC content.

The chloroplast genome size ranges from 139,500 to 139,801 bp, with all samples consistently displaying a GC content of 38.9%. All chloroplast genomes exhibit a typical quadripartite structure (**Figure 1**), consisting of a long single-copy region (LSC), a short single-copy region (SSC), and two internal inverted repeat regions (IRA and IRB). The length of the LSC region ranges from 83,007 bp (*G. zixingensis*) to 83,364 bp (*G. subsolidus* GDHZ), while the SSC varies from 12,799 bp (*G. subsolidus* GXLB) to 12,882 bp (*G. kunsishii*). The lengths of the inverted repeat regions range from 21,796 bp (*G. dongdingensis*) to 21,842 bp (*G. multifolius* and *G. zixingensis*). Across all genomes, a total of 137 genes were identified, including 90 protein-coding genes, 39 tRNA genes, and eight rRNA genes (**Supplementary Tables S3**, **S4**).

For nrDNA repeats, the combined length of 27 tandem repeat sequences ranges from 5,802 to 5,804 bp. The 18S region is constant at 1,813 bp, the ITS1 region varies between 213 bp and 214 bp, the 5.8S region remains constant at 160 bp, the ITS2 region ranges from 233 bp to 234 bp, and the 28S region varies between 3,382 bp and 3,384 bp (**Supplementary Figure S2**).

A total of 29 mitochondrial protein-coding genes were identified, among which 22 were complete, and seven were incomplete. With the exception of the *rps*1 (*ribosomal protein S1*) and *cyt*b (*cytochrome b*) genes, the recovery rates of the remaining genes were low (**Supplementary Figure S2**).

### 3.2 Phylogenetic reconstruction

A total of four alignment matrices for phylogenetic analysis were generated from aligned sequences after trimming nonconserved regions: (1) 75 complete chloroplast genomes, totaling 139,357 bp; (2) 75 chloroplast genomes with the inverted repeat region removed, totaling 117,306 bp; (3) complete nrDNA repeat sequences, totaling 5,805 bp; (4) 24 shared mitochondrial genes from 27 samples, totaling 19,727 bp (**Supplementary Data Sheets 1**–**4**).

**Figure 2:**
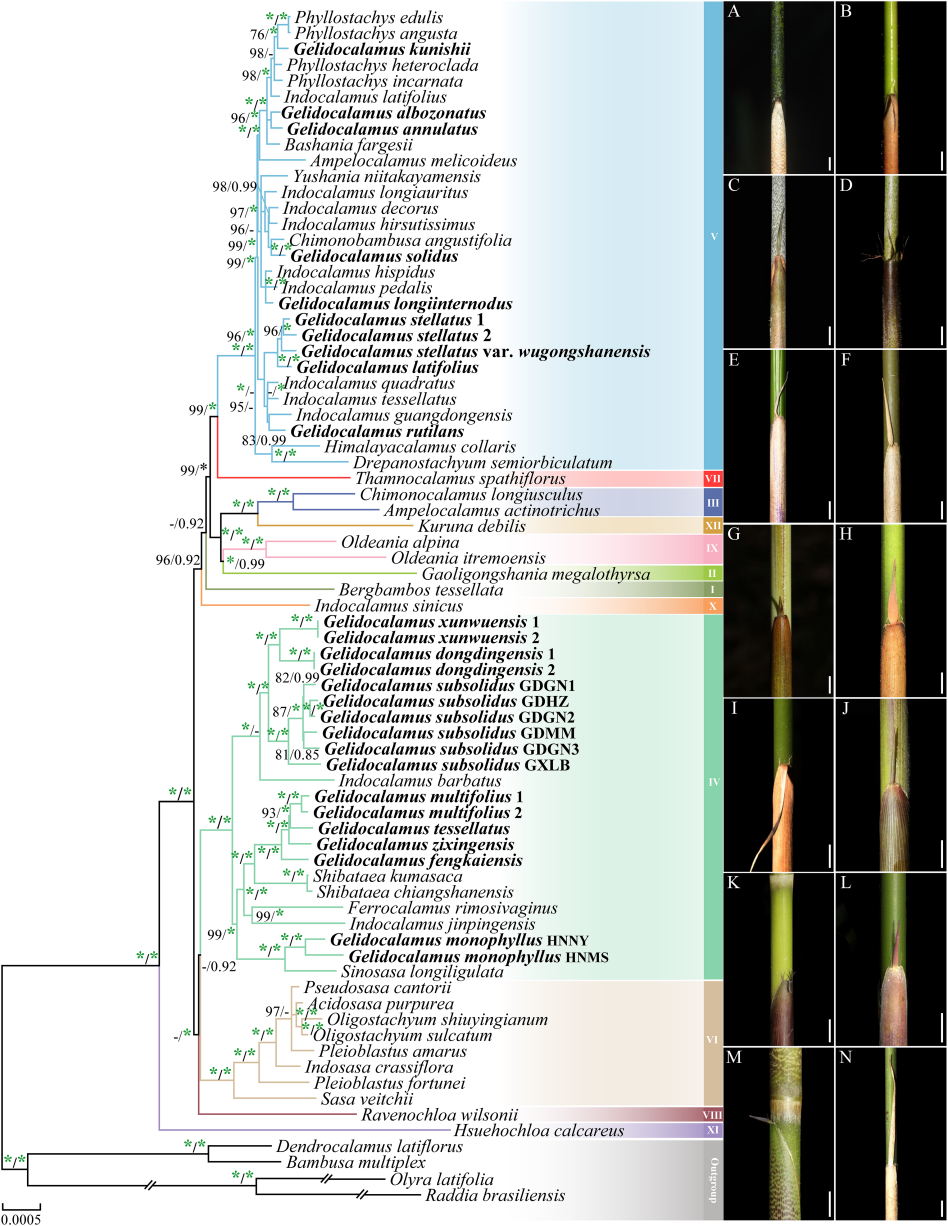
Phylogenetic tree of 75 taxa using maximum likelihood (ML) and Bayesian inference (BI) methods based on plastomes. ML tree topology is shown with ML bootstrap values, and BI posterior probabilities are indicated on the nodes. Only bootstrap values (BS) ≥ 75% and posterior probabilities (PP) ≥ 0.75 are indicated at each node; otherwise, dashes are used. The green asterisk indicates support of 100% BS or 1.00 PP. **(A–N)** represent the characteristics of the culms and culm sheaths of 14 species of *Gelidocalamus*. **(A)** *G. albozonatus*, **(B)** *G. annulatus*, **(C)** *G. solidus*, **(D)** *G. longiinternodus*, **(E)** *G. stellatus*, **(F)** *G. latifolius*, **(G)** *G. rutilans*, **(H)** *G. xunwuensis*, **(I)** *G. dongdingensis*, **(J)** *G. subsolidus*, **(K)** *G. multifolius*, **(L)** *G. zixingensis*, **(M)** *G. Tessellatus*, and **(N)** *G. monophyllus*.

Based on the matrices of the complete chloroplast genome and the chloroplast genome with the inverted repeat region removed, the reconstructed phylogenetic relationships using maximum likelihood and Bayesian inference methods exhibit largely consistent topologies (**Figure 2**; **Supplementary Figure S3**). The Arundinarieae tribe emerges as a strongly supported monophyletic branch (BSML = 100% and PPBI = 1.00), with its twelve major subclades also receiving robust support. Members of the *Gelidocalamus* are grouped into two distinct monophyletic clades (IV and V), clustering with closely related genera such as *Indocalamus*, *Shibataea*, and *Ferrocalamus*.

In clade V, there are a total of 10 *Gelidocalamus* samples, comprising eight species and one variety. Among them, *G. stellatus*, *G. stellatus* var. *wugongshanensis* and *G. latifolius* (BSML = 96% and PPBI = 1.00), as well as *G. albozonatus* and *G. annulatus* (BSML = 96% and PPBI = 1.00), each form two distinct monophyletic clades, respectively. The remaining species are distributed throughout the clade and cluster separately with the genera *Indocalamus* and *Phyllostachys*. In clade IV, *G. xunwuensis*, *G. dongdingensis*, and *G. subsolidus* form a monophyletic clade, and cluster with *Indocalamus barbatus* as a sister group (BSML = 100% and PPBI = 0.66). Meanwhile, *G. multifolius*, *G. tessellatus*, *G. zixingensis*, and *G. fengkaiensis* form a monophyletic group with two *Shibataea* species as a sister group (BSML = 100% and PPBI = 1.00). Additionally, *G. monophyllus* and *Sinosasa longiligulata* cluster together in a clade (BSML = 100% and PPBI = 1.00).

Compared to chloroplast genome data, the phylogenetic relationships based on nrDNA repeats sequences and mitochondrial genes showed weak support and exhibited significant topology differences between the two datasets, both displaying multiple clades (**Supplementary Figures S4**, **S5**).

### 3.3 Divergence time estimation

**Figure 3:**
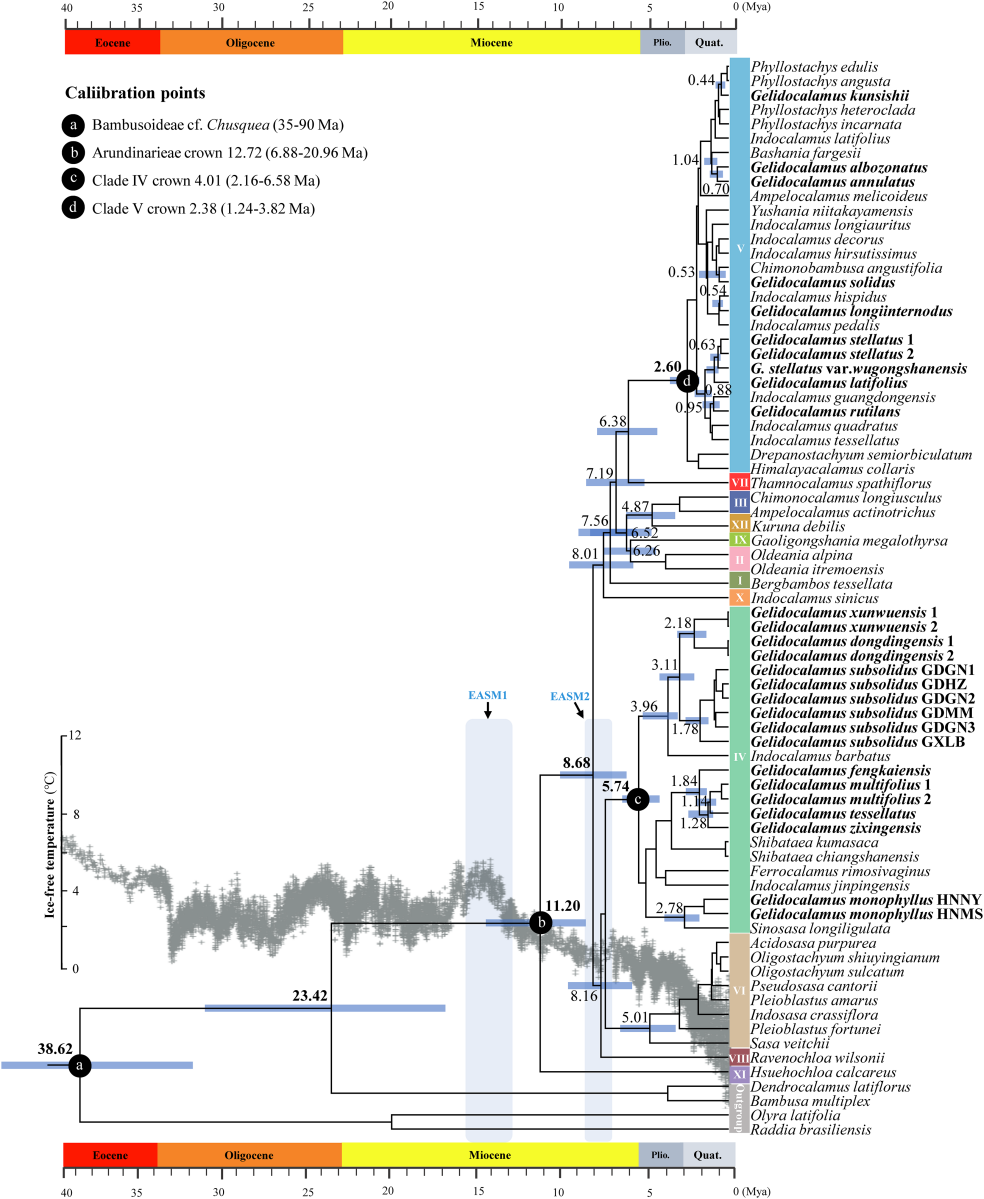
Divergence times of major clades in *Gelidocalamus*. The chronogram of the *Gelidocalamus* and relatives is based on BEAST analysis of plastomes DNA. Four calibration points are marked by red circles. The ice-free temperature change curves over the last 40 million years as modified in Zachos et al. (2008). Blue characters and shades of light blue indicate the approximate periods of two intensification of the East Asian monsoon climate. Node bars represent the 95% highest posterior density intervals for node ages, with the mean ages of some important nodes in Arundinarieae indicated.

Estimates of divergence times indicate that the stem age of the Arundinarieae tribe diverged in the Late Oligocene (23.42 Mya, 95% HPD: 16.71–32.21 Mya) and the crown age diverged around 11.20 Mya (95% HPD: 8.22–14.59 Mya) in the Mid–Late Miocene (**Figure 3**). The crown age for clades IV and V, where *Gelidocalamus* is situated, was 5.74 Mya (95% HPD: 4.50–6.58 Mya) and 2.60 Mya (95% HPD: 1.80–3.56 Mya), respectively. The earliest divergence of *Gelidocalamus* species, including *G. subsolidus* and *G. xunwuensis*, from their common ancestor with *Indocalamus barbatus* occurred at 3.96 Mya (95% HPD: 2.71–5.01 Mya). In contrast, *G. kunsishii* was the latest to diverge at 0.44 Mya (95% HPD: 0.24–0.69 Mya). All other members of *Gelidocalamus* appeared successively within the past three million years (**Supplementary Table S5**).

### 3.4 Genome structural variations and sequence divergences

The results of phylogenetic inference indicate that *Gelidocalamus* is a polyphyletic group, with its members intermingled with closely related genera, forming clades IV and V. Therefore, this study provides insights into the structure and evolution of the chloroplast genomes of *Gelidocalamus* and its close relatives within the two clades.

**Figure 4:**
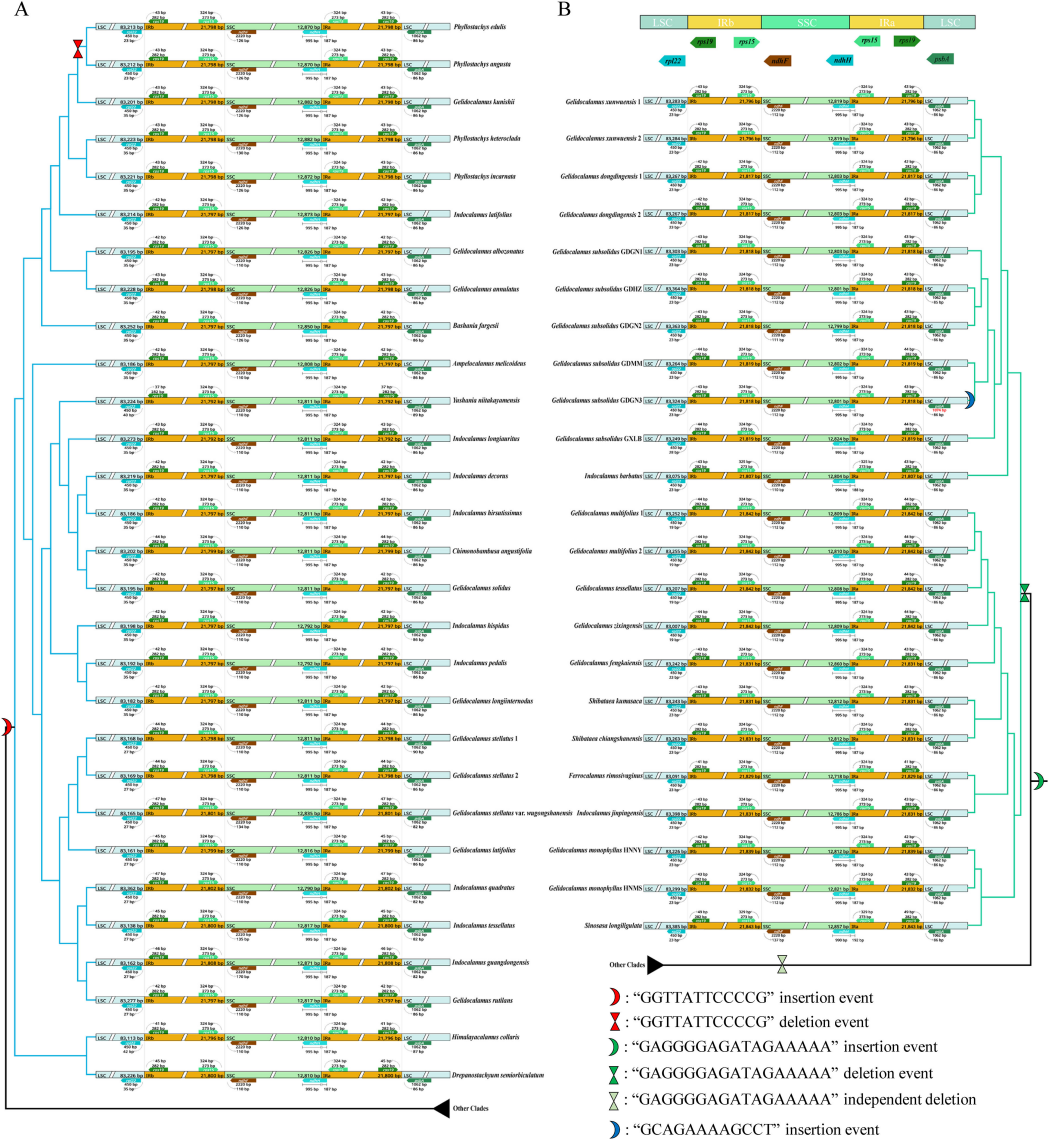
Visualization of the expansion and contraction in the inverted repeat region (IR) boundary of chloroplast genomes. The topology is based on the ML tree using complete plastomes. **(A)** Clade V; **(B)** clade IV.

The collinearity analysis results based on Mauve indicate the absence of any detected rearrangements or inversions within these two clades (**Supplementary Figure S6**). The boundary expansion and contraction analysis revealed that six genes located at the junctions, namely *rpl*22, *rps*19, *rps*15, *ndh*F, and *ndh*H, exhibited consistent lengths across all samples, except for *psb*A, which showed variations in length in *G. subsolidus* GDGN3. Only the *ndh*H gene spans the boundaries between the SSC/IRa regions. In the boundary comparison analysis, we identified an insertion event specific to clade V. The insertion sequence “GGTTATTCCCCG” at the LSC/IRb junction represents an insertion event before the most recently common ancestor of clade V but was later lost in the ancestor of two *Phyllostachys* species (*P. edulis* and *P. angusta*) (**Figure 4A**; **Supplementary Figure S7**). Another insertion sequence “GAGGGGAGATAGAAAAA”, located at the SSC/IRa junction, is present in many subclades of the Arundinarieae tribe but has been lost to varying degrees across them. Notably, all species in clade IV entirely lack this sequence (**Figure 4A**; **Supplementary Figure S8**). Additionally, in clade IV, a species-specific borderline insertion “GCAGAAAAGCCT” at the LSC/IRa junction was identified in the plastome of *G. subsolidus* GDGN3 (**Figure 4B**; **Supplementary Figure S9**).

**Figure 5:**
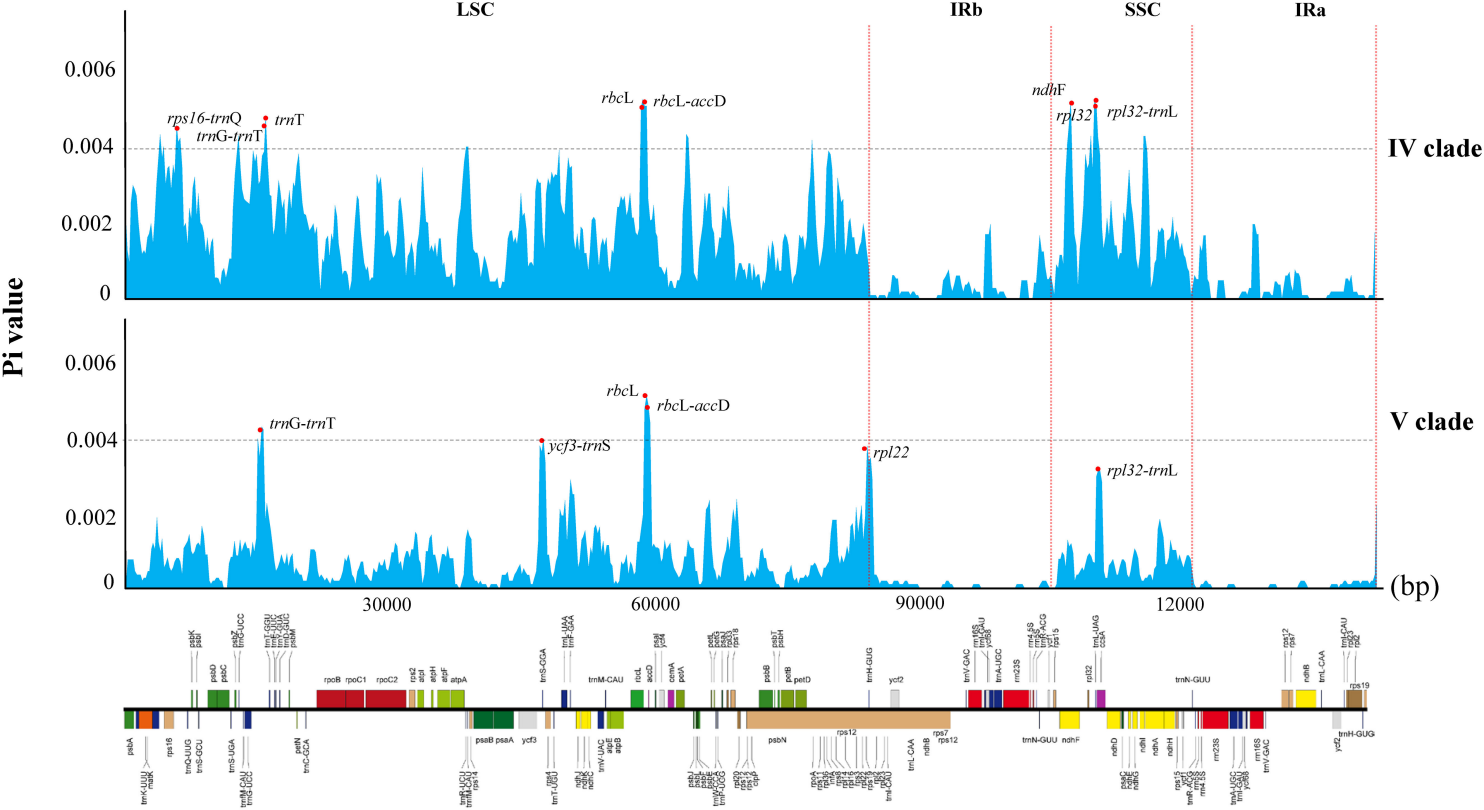
Comparative analysis of nucleotide variability by pi values of the two clades presented in a sliding window (window length: 600 bp; step size: 200 bp).

The nucleotide diversity (pi) of the two clades ranges from 0 to 0.005 in clade V and 0 to 0.00598 in clade IV, with average pi values of 0.0016 and 0.0006, respectively. Notably, the average pi value for the SSC region exceeds that of the LSC region, while the inverted repeat region exhibits the lowest nucleotide diversity in both clades (**Figure 5**). Among the variable sites, clade IV has 27 sites with a π value exceeding 0.004, whereas clade V has only three such sites. As shown in **Figure 5**, the highly variable regions in the clade IV include intergenic regions such as *trn*K-*rps*16, *trn*G-*trn*T, *rbc*L-*acc*D, and *rpl*32-*trn*L, with *rbc*L-*acc*D region exhibiting the highest pi value (0.00598). Coding genes such as *ndh*F, *rpl*32, and *trn*T also show high variability, with *ndh*F having the highest pi value (0.00595). In clade V, only three loci exceed a pi value of 0.004: *rbc*L, *rbc*L-*acc*D, and *trn*G-*trn*T, with the *rbc*L gene locus showing the highest pi value (0.005).

### 3.5 Codon usage bias

**Figure 6:**
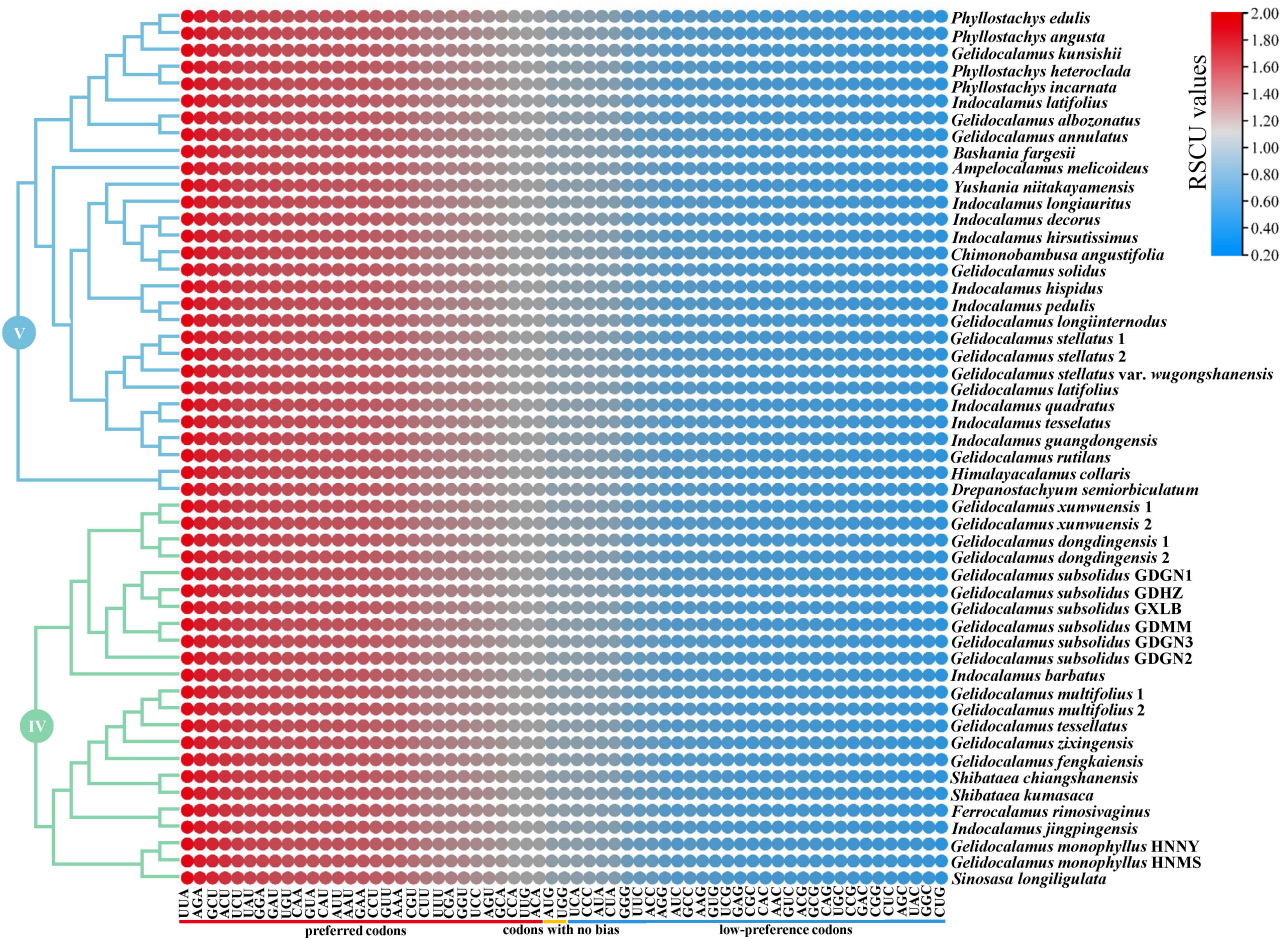
The relative synonymous codon usage (RSCU) values of all merged CDS for the two clades. The red values indicate higher RSCU values, and the blue values indicate lower RSCU values.

The codon usage bias of clades IV and V is illustrated in **Figure 6**. A total of 29 preferred codons (RSCU > 1), 30 low-preference codons (RSCU < 1), and two codons showing no bias (RSCU = 1) were detected in clade V. However, in *Drepanostachyum semiorbiculatum* (clade V) and *G. monophyllus* HNNY (clade IV), 29 low-preference codons and three codons with no codon usage bias were identified. Among the 29 preferred codons, only two codons (UUG/UCC) end with C/G, while the rest end with A/U. The total codon usage among the IV clade chloroplast genomes ranges from 20,750 to 20,893 codons (average 20,866), and from 20,777 to 20,877 codons (average 20,870) among the V clade. Notably, the amino acid leucine, encoded by UUA, UUG, CUU, CUC, CUA, and CUG, has the highest frequency of usage, ranging from 2,251 to 2,269 codons. In contrast, the amino acid cysteine, encoded by UGU and UGC, has the lowest frequency of usage, ranging from 229 to 233 codons.

### 3.6 SSRs and long repeats analysis

**Figure 7:**
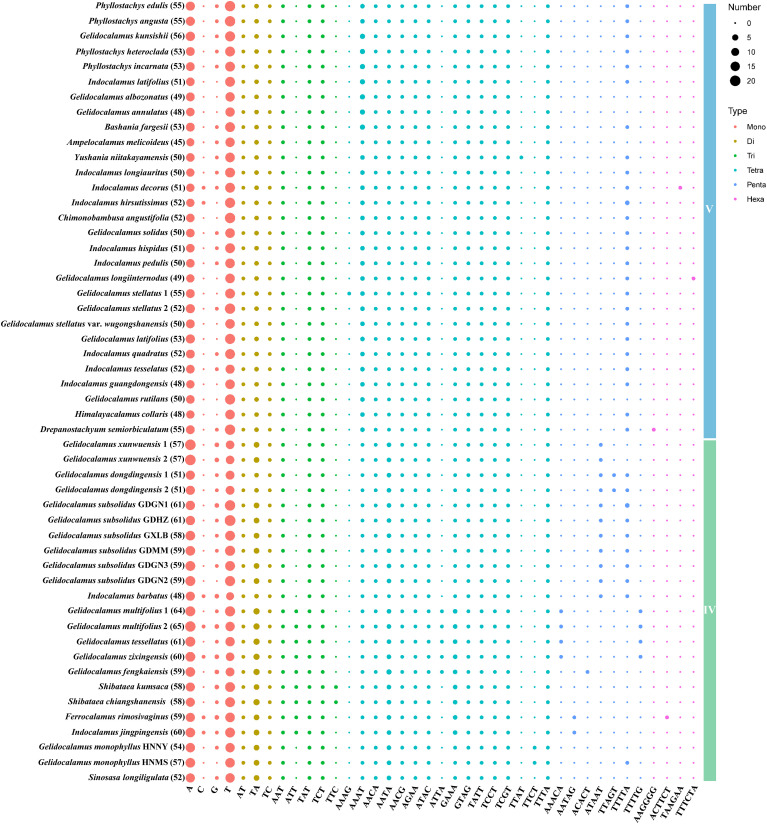
Plot of SSR repeat pattern numbers in two clades. The number following each species name indicates the total number of SSRs in each plastome.

A total of 1,485 SSRs were identified in clade V and 1,328 in clade IV. The number of SSRs varied across species, ranging from 45 in *Ampelocalamus melicoideus* to 56 in *G. kunsishii* within clade V, and from 48 in *Indocalamus barbatus* to 65 in *G. multifolius* 2 within clade IV. In both clades that include *Gelidocalamus* members, mononucleotide repeats were the most abundant type of SSRs, followed by tetranucleotide repeats. Conversely, hexanucleotide repeats were the least frequent, found only in three species in each clade: *I. decorus*, *G. longiinternodus*, and *Drepanostachyum semiorbiculatum* in clade V; and *Ferrocalamus rimosivaginus*, *I. jingpingensis*, and *G. monophyllus* HNMS in clade IV. As shown in **Figure 7**, among the 868 mononucleotide repeats in clade V, A/T repeats were predominant (97.8%), whereas C/G repeats were much less common (2.2%). Similarly, in clade IV, A/T repeats constituted a high percentage (95.3%) of the 759 mononucleotide repeats. Among the 118 dinucleotide repeats in clade V, TA repeats were the most abundant (49.1%), with AT and TC repeats being approximately equal (around 25%). In clade IV, TA repeats accounted for 66.6%, while AT and TC repeats were about 16.6% each. The number of species with other repeat types did not significantly differ between the two clades. Trinucleotide repeats in both clades were predominantly AAT, TAT, and TCT. Among tetranucleotide repeats, except for less frequent types such as AAAG, ATTA, TTAT, and TTCT, the quantities of other types were relatively similar. TTTTA was the most prevalent pentanucleotide repeat, and only three occurrences of hexanucleotide repeats were identified in each clade.

The distribution of SSRs within the chloroplast genomes varied across different regions, with the highest density found in the LSC region and fewer in the SSC and IR regions. The distribution also varied across intron, CDS, and IGS regions, with the IGS region containing the largest number of SSRs. The proportion of distribution across different regions was relatively consistent among species (**Supplementary Figures S10**-**S12**). Additionally, we identified 1,907 and 1,647 long repeats in clades V and IV, respectively. Forward repeats were the predominant type of long repeat. Similar to SSRs, the distribution of long repeats is primarily concentrated in the LSC region. However, unlike SSRs, the distribution of long repeats showed that the highest number was found in the CDS region, followed by the IGS region, with the intron region having the fewest (**Supplementary Figures S13**-**S15**).

## 4 Discussion

### 4.1 Assembly efficiency of different molecular markers from genome skimming data of *Gelidocalamus*

Previous studies have utilized low-depth genomic sequencing data to capture highly copied chloroplast genomes within the total genomic DNA, while the effectiveness of other molecular markers, such as mitochondrial genome and nuclear ribosomal DNA repeats has been limited. Recent studies indicated that the organelle genome data comprises only a small portion of the total genomic DNA (Straub et al., 2012). By increasing the sequencing depth to around 10X, it is possible to retrieve complete chloroplast, mitochondrial genomes, nrDNA repeats, and a substantial number of single-copy nuclear genes (SCNs) from genome skimming data (Vargas et al., 2019; Liu et al., 2021). To evaluate the feasibility of retrieving complete mitochondrial genome data and nuclear ribosomal DNA repeats from low-depth genome skimming data (2-4 X coverage) in bamboo, we performed assemblies using the sequencing data generated in this study. The results indicated successful retrieval of complete chloroplast genomes and nrDNA repeats, although the reconstruction of the mitochondrial genome remained incomplete. A total of 29 conserved mitochondrial protein-coding genes were identified. Previous studies have shown that a sequencing depth of less than 5X coverage is sufficient to assemble the complete chloroplast genome, some mitochondrial coding genes and the entire nrDNA repeats (Straub et al., 2012; Fonseca and Lohmann, 2020). For the Arundinarieae tribe, it has been reported that approximately 2G of data (1X coverage) is adequate for assembling the complete chloroplast genome and nrDNA repeats, which aligns with our findings. Consequently, in bamboo plants, the complete chloroplast genome and nrDNA repeats can still be assembled with shallower sequencing coverage. Additionally, our study found that approximately two times coverage was adequate to assemble partially complete mitochondrial coding genes and entire nrDNA repeats. However, low-coverage genomic data proved insufficient for successfully assembling single-copy nuclear genes (Guo et al., 2021).

### 4.2 Phylogenetic relationship of *Gelidocalamus*

The incorporation of new molecular evidence into analyses has progressively refined the framework of phylogenetic relationships within the Arundinarieae tribe and its major clades. Previous studies, however, involved only a limited number of *Gelidocalamus* to represent the genus, leaving the internal phylogenetic relationships within *Gelidocalamus* unclear (Triplett and Clark, 2010; Zeng et al., 2010; Yang et al., 2013; Attigala et al., 2014; Zhang et al., 2020; Guo et al., 2021). Our study utilized extensive sampling, encompassing all 16 species and one variety of *Gelidocalamus*, totaling 27 samples from diverse populations. Representative samples from closely related genera were included to unravel both intrageneric and intergeneric relationships within the *Gelidocalamus*. The results based on chloroplast genomes strongly support the monophyly of the Arundinarieae tribe (BSML = 100% and PPBI = 1.00), which is divided into twelve well-supported clades. *Gelidocalamus* species are distributed across two of these clades (clades IV and clades V) with high support (BSML = 100% and PPBI = 1.00). This research achieved enhanced resolution compared to previous studies with low support values at various nodes. The findings indicate that species such as *G. tessellatus*, *G. xuwuensis*, *G. rutilans*, *G. latifolius*, and *G. stellatus* are distributed across two distinct clades, consistent with previous research (Ma et al., 2017; Guo et al., 2019; Zhang et al., 2019). Species not included in previous studies are also found within these two clades, and the phylogenetic relationships are strongly supported. These results affirm the polyphyletic nature of the *Gelidocalamus*, challenging traditional morphological classifications. For instance, *G. stellatus*, despite its morphological similarity to *G. tessellatus* and *G. xuwuensis*, belongs to a separate clade. Additionally, species with significant morphological differences, such as *G. longiinternodus*, which exhibit traits like sickle-shaped auricles, single branch on one-year-old plants, and spring sprouting (Wen, 1986), which display characteristics typical of *Indocalamus*. Molecular evidence supports a close relationship between *G. longiinternodus* and the *Indocalamus* genus. The classification boundaries of *Gelidocalamus* remain contentious and require further revision.

The use of different molecular markers to infer phylogenetic relationships often leads to controversies. For instance, Peng et al. (2008) found that *G. stellatus* is closely related to *Ferrocalamus strictus* based on nuclear single-copy genes *GBSS*I and ITS, contrasting sharply with results based on complete plastid genomes, indicating significant incongruence between nuclear and plastid. Similar conflicts have been observed in previous research, Guo et al. (2021) investigated the phylogenetic relationships within the Arundinarieae tribe using ddRAD data, nrDNA sequences, and complete chloroplast genomes. Their results indicated that the phylogenetic relationships inferred from ddRAD data closely resembled those derived from nrDNA sequences, with *Gelidocalamus* consistently grouped within the “*Indocalamus* + *Gelidocalamus* + *Chimonocalamus*” clade. Most *Gelidocalamus* species formed a monophyletic branch, which aligns with the “core species of *Gelidocalamus*” (Li et al., 2024). However, the limited sampling of species for nrDNA sequences and chloroplast genomes restricted comprehensive comparisons across the different molecular datasets. Nevertheless, the phylogenetic relationships based on the chloroplast genome corroborate previous findings, although they exhibit significant conflict with those derived from nuclear genes. Potentially due to factors such as hybridization (Yang et al., 2013), incomplete lineage sorting, and chloroplast capture (Maddison, 1997; Peng et al., 2008; Triplett and Clark, 2010; Zhang et al., 2012; Ma et al., 2017; Guo et al., 2019, Guo et al., 2021). We attempted to establish intrageneric phylogenetic relationships using complete nrDNA repeats and 24 shared mitochondrial protein-coding genes from each species. However, the results indicated that these highly conserved sequences provided limited informative sites, which impeded the construction of a clear phylogenetic framework. In some instances, the variation within species exceeded that between species (**Supplementary Figures S4**, **S5**), likely due to incomplete concerted evolution and pseudogene phenomena affecting ITS sequences in temperate woody bamboos (Gernandt et al., 2001; Wei et al., 2003). The use of different molecular markers introduced variability in phylogenetic relationships, adding complexity to the phylogenetic inference of *Gelidocalamus*. The observed conflict between nuclear and plastid genes suggests that these datasets may have experienced divergent evolutionary histories. Integrating morphological taxonomy with the ddRAD data, we propose that the phylogenetic relationships inferred from ddRAD sequencing offer a more robust framework for understanding the phylogeny of *Gelidocalamus*. This study has elucidated the phylogenetic relationships of *Gelidocalamus* using complete chloroplast genomes and assessed the utility of other molecular markers. However, for complex taxa with reticulate evolutionary histories, such as *Gelidocalamus*, phylogenetic resolution may not be fully achieved through molecular data alone. The morphological challenges associated with their taxonomy are equally complex. Therefore, a comprehensive approach that integrates both morphological features and molecular evidence is crucial for gaining deeper insights into their classification and evolutionary history.

### 4.3 Evolution history of *Gelidocalamus*

To investigate the origin and evolution of *Gelidocalamus*, we used data from complete chloroplast genomes to estimate divergence times. The findings reveal that the crown-group divergence time of the Arundinarieae tribe, to which *Gelidocalamus* belongs, is approximately in the Late Miocene, around 11.20 Mya (95% HPD: 8.22–14.59 Mya). Our estimated divergence time is slightly younger than those reported in earlier studies, such as 18.81 Mya (Christin et al., 2013) and 18.73 Mya (Guo et al., 2021), but older than 8.96 Mya (Bouchenak-Khelladi et al., 2010) and 10.01 Mya (Hodkinson et al., 2010), and closely aligned with 12.72 Mya (Zhang et al., 2016). The results suggest that *Gelidocalamus* is a relatively young genus, with all its members having differentiated within a short period of time. However, chloroplast genome data indicate that *Gelidocalamus* has a nonmonophyletic origin.

Previous studies have indicated that the Arundinarieae experienced a period of rapid radiation and differentiation during the Mid- or Late Miocene (Zhang et al., 2016; Guo et al., 2021). Our results align with those of Zhang et al. (2016), showing that the 12 major clades began to diverge rapidly in the Late Miocene. This rapid divergence is commonly linked to significant climate changes (Janssens et al., 2009; Hampe and Jump, 2011; Zou et al., 2013), particularly evident during two intensification of the East Asian monsoon (EASM) climate around 15.5–13 Mya and 8–7 Mya (Zhisheng et al., 2001; Zheng et al., 2004; Zhang et al., 2016). The cooler temperatures and increased summer rainfall during these periods created favorable conditions for temperate woody bamboos. These bamboos swiftly colonized moist slopes, establishing themselves as dominant species. Their effective underground rhizome development further supported their rapid spread and diversification. Previous studies have suggested that incomplete lineage sorting (ILS) may be prevalent in the Arundinarieae tribe due to rapid radiative divergence, which led to species differentiation within a short time frame. This rapid divergence resulted in significant morphological changes that outpaced genetic diversification, leading to substantial morphological differences among species while they remain closely related genetically. Additionally, overlapping distributions and potential hybridization among species may have further complicated the phylogenetic relationships within the genus *Gelidocalamus*. Given the limitations of the dataset used in this study, these issues may require further investigation (Zhang et al., 2012; Yang et al., 2013; Guo et al., 2019, Guo et al., 2021).

### 4.4 The diversity of plastome characteristics

Chloroplasts, the primary site of photosynthesis, are crucial for plant physiological and developmental processes (Verma and Daniell, 2007; Li et al., 2020). In angiosperms, chloroplast genomes are mostly maternally inherited and highly conserved, with moderate substitution rates. Recently, chloroplast genomes have been widely used for plant phylogenetic studies (Zhang and Li, 2011; Wang et al., 2012; Cui et al., 2023; Peng et al., 2023).

To investigate the evolution of the chloroplast genome in *Gelidocalamus*, we conducted comparative analyses among species within the genus and its close relatives across the two clades in which it is distributed. The gene order and gene content are uniform across all chloroplast genomes. No significant expansion or contraction phenomena were detected at the SSC/IR region boundaries, nor were structural rearrangements or inversion events observed. Insertion events were observed at the boundaries of clades V and IV, with varying degrees of loss and new insertions occurring across different clades. Despite these variations, the overall similarity between the two clades remains high. The expansion and contraction of regions can largely be attributed to these insertions and deletions.

Synonymous codon preference, a crucial feature influencing gene expression and biological evolution, is impacted by variations and natural selection (Duret, 2000; Romero et al., 2000; Duret, 2002; Yuan et al., 2021). In this study, 29 preferred codons were identified among the protein-coding genes of two clades. Only two codons (UUG/UCC) ended with C/G, while the rest ended with A/U, consistent with previous research indicating a high level of consistency in codon preference and usage patterns within the subfamily Bambusoideae (Li et al., 2019). SSRs, a class of short-sequence repeat units widely distributed across genomes, are excellent genetic markers due to their polymorphic nature and high mutation rates (Cavalier-Smith, 2002; Yang et al., 2013; Asaf et al., 2018). In this study, we identified SSRs and long repeats within the two clades containing *Gelidocalamus*. The results show that *Gelidocalamus* exhibits minimal variation compared to closely related genera within the same clade. However, substantial differences in the number of SSRs and long repeats were observed between congeneric species across different clades.

DNA barcoding employs standardized, sufficiently variable, easily amplifiable, and relatively short DNA sequences for species identification (Hebert et al., 2003; Kress et al., 2005). Various molecular barcodes have been developed for identifying species within the subfamily Bambusoideae (Zhang and Li, 2011). In this study, we identified 17 hypervariable regions (pi > 0.004) in clade IV, including *trn*K-*rps*16, *trn*G-*trn*T, *rbc*L-*acc*D, *rpl*32-*trn*L, *rbc*L, and *rpl*32. Conversely, only three hypervariable regions (*rbc*L, *rbc*L-*acc*D, and *trn*G-*trn*T) were identified from clade V. Many of these identified highly variable loci have been previously used as molecular markers for rapid identification and phylogenetic analysis (Zhang and Li, 2011), demonstrating their stability for plant identification. However, variability among species within the two clades containing *Gelidocalamus* differs, suggesting that the degree of species differentiation within this genus is not uniform across the chloroplast genomes. This variability may contribute to the separation into two major clades and reflects similar patterns observed in related genera, underscoring the complexity of the phylogenetic relationships among bamboo species.

## 5 Conclusions

In this study, all species and varieties of *Gelidocalamus* were collected (except for *G. kunishii* and *G. fengkaiensis*, which lacked morphological survey records). A total of 27 chloroplast genomes were assembled from genome skimming data for 16 species and one variety within *Gelidocalamus*. Additionally, complete nrDNA repeats and 29 mitochondrial protein-coding genes shared by each species were assembled. Through extensive sampling, we reconstructed high-resolution intrageneric and intergeneric phylogenetic relationships within *Gelidocalamus* based on complete chloroplast genome data. However, the phylogenetic relationships based on nrDNA repeats and mitochondrial genes exhibited extremely low resolution. Estimations of Divergence times of *Gelidocalamus* suggested it to be a young genus. The cause of intragenomic polyphyly may be linked to a rapid radiation event experienced by the Arundinarieae tribe. Furthermore, a comparative analysis of chloroplast genome structure and variation was conducted on the two clades involving *Gelidocalamus* species. The results revealed a high degree of consistency among all chloroplast genomes in terms of structural collinearity, gene count, gene order, and synonymous codon preference. This study comprehensively elucidated the phylogenetic relationships within *Gelidocalamus* for the first time, estimated its evolutionary times, and compared structural differences in various chloroplast genomes. However, conflicts arising from different molecular markers, such as nuclear genes and plastid genomes, may require further evidence and in-depth exploration.

## Data Availability

The sequence data have been submitted to the GenBank databases under accession numbers OP920757-OP920759, and PP999719-PP999742.

## Supplementary material

Supplementary Data Sheet 1 The alignment of 75 complete chloroplast genomes.

Supplementary Data Sheet 2 The alignment of 75 complete chloroplast genomes with the inverted repeat region removed.

Supplementary Data Sheet 3 The alignment of nrDNA repeat sequences.

Supplementary Data Sheet 4 The alignment of 24 shared mitochondrial genes from 27 samples.
