# Supplementary material for: Comparative analysis of chloroplast genomes and phylogenetic relationships in the endemic Chinese bamboo Gelidocalamus (Bambusoideae)
Source: Front Plant Sci. 2024 Nov 11;15:1470311. doi: 10.3389/fpls.2024.1470311 (PMC11586178; doi:10.3389/fpls.2024.1470311)
Supplement: Supplementary file 5 [file DataSheet5.pdf]

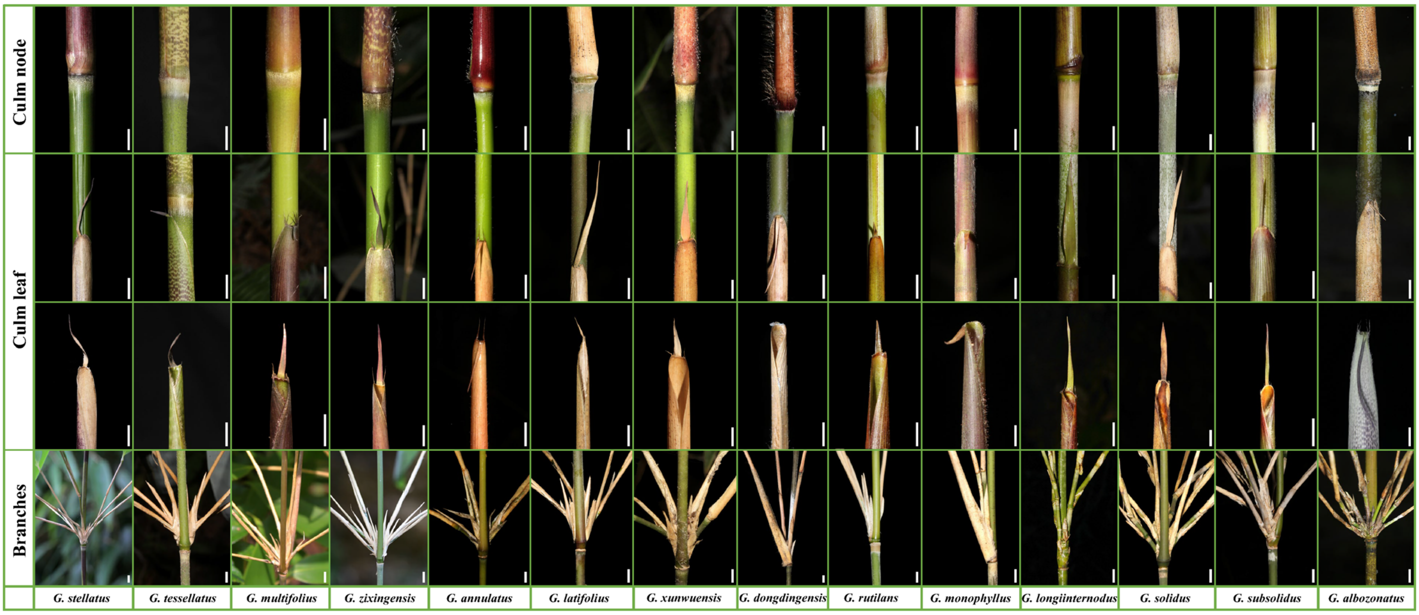

Supplementary Figure 1 The main morphological characteristics of the *Gelidocalamus* (lacking *G. stellatus* var. *wugongshanensis*, *G. fengkaiensis* and *G. kunishii*). Scale bars: 10 mm

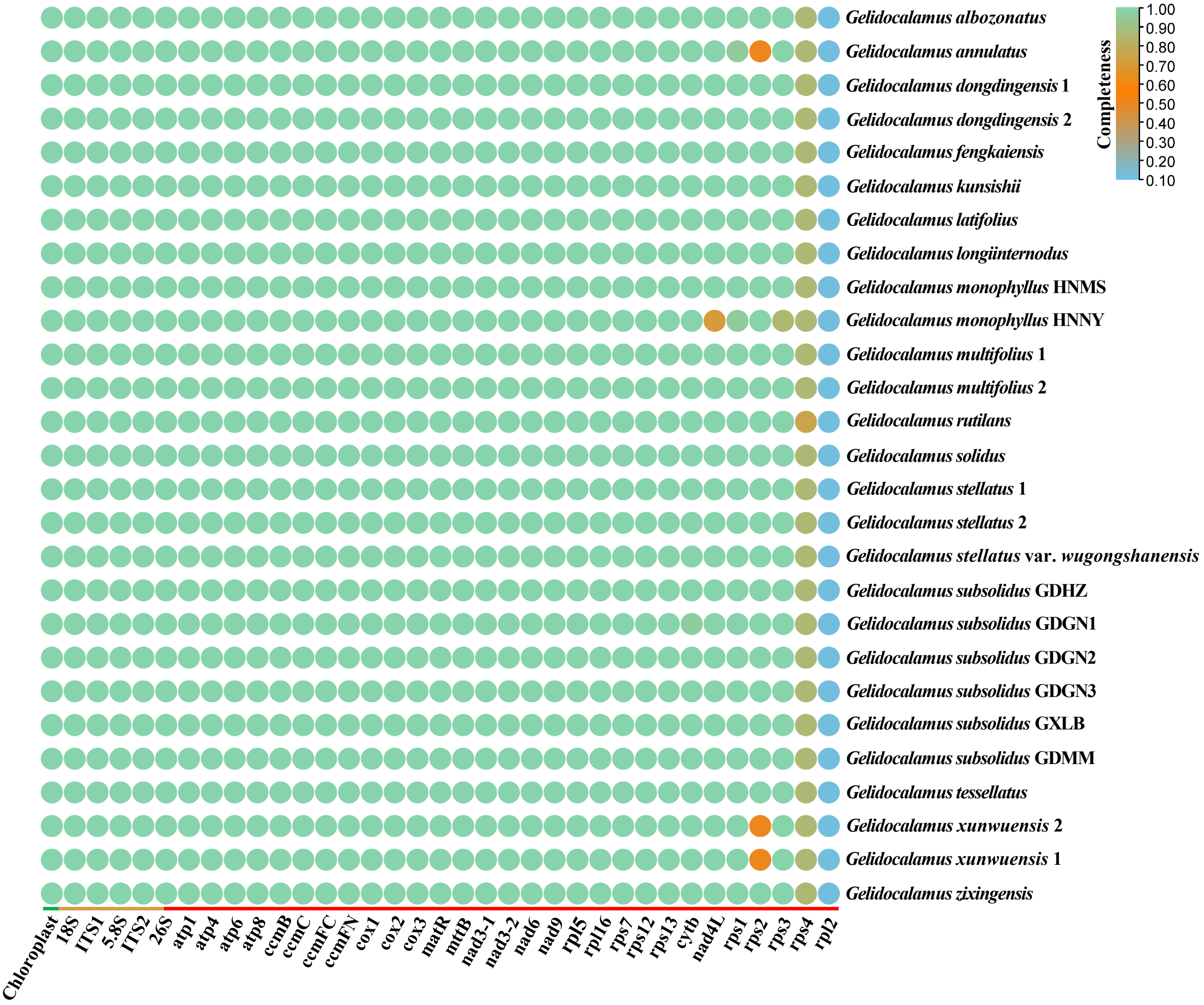

Supplementary Figure 2 Assembly results of chloroplast genome, nrDNA repeats, and mitochondrial genes. Different colors represent assembly completeness, ranging from green (high completeness) to blue (low completeness).

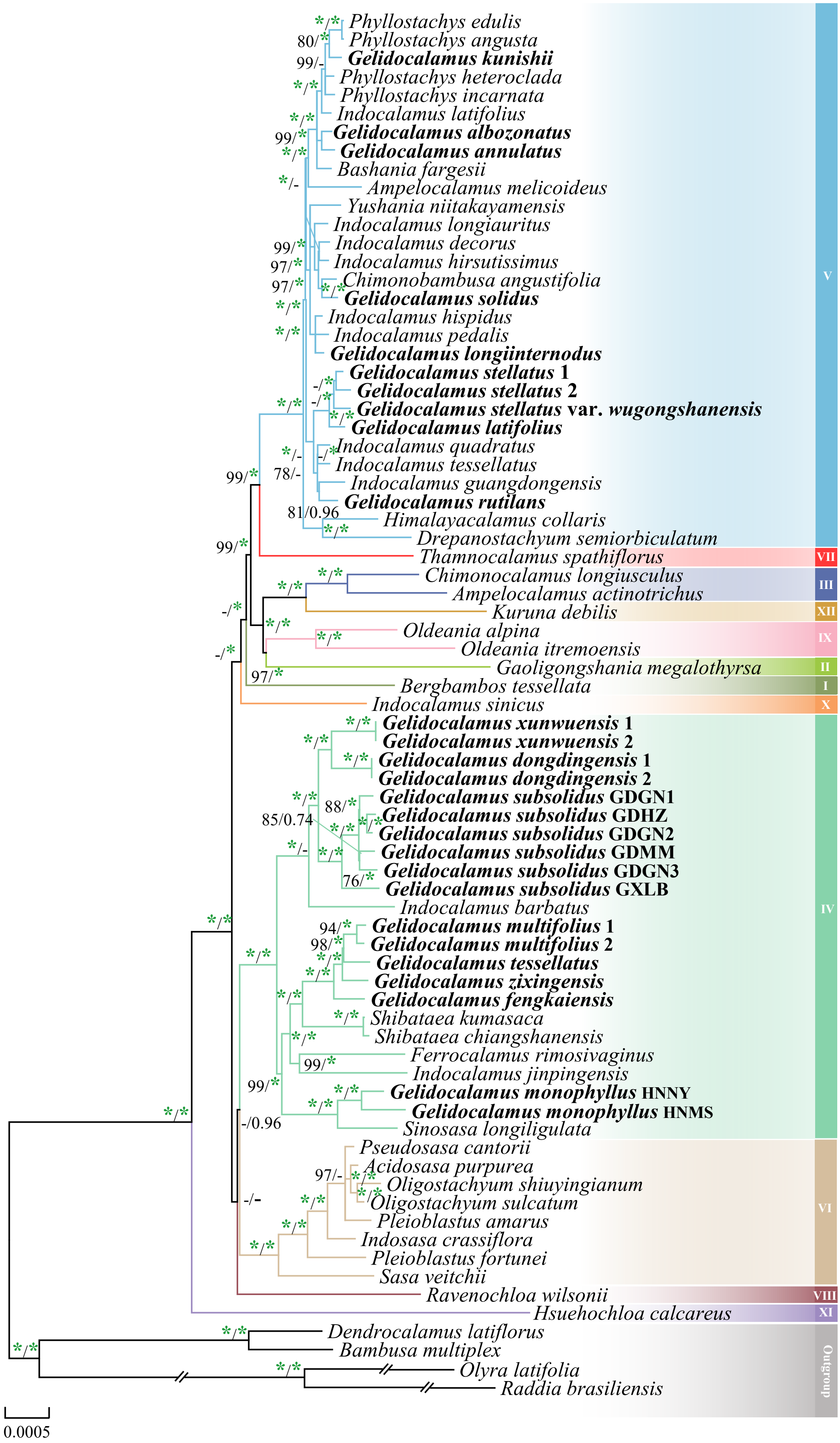

Supplementary Figure 3 Phylogenetic tree of 75 taxa using maximum likelihood (ML) and Bayesian inference (BI) methods based on plastomes with one IR excluded. Tree reconstruction methods and percent bootstrap values are as described in Figure 3.

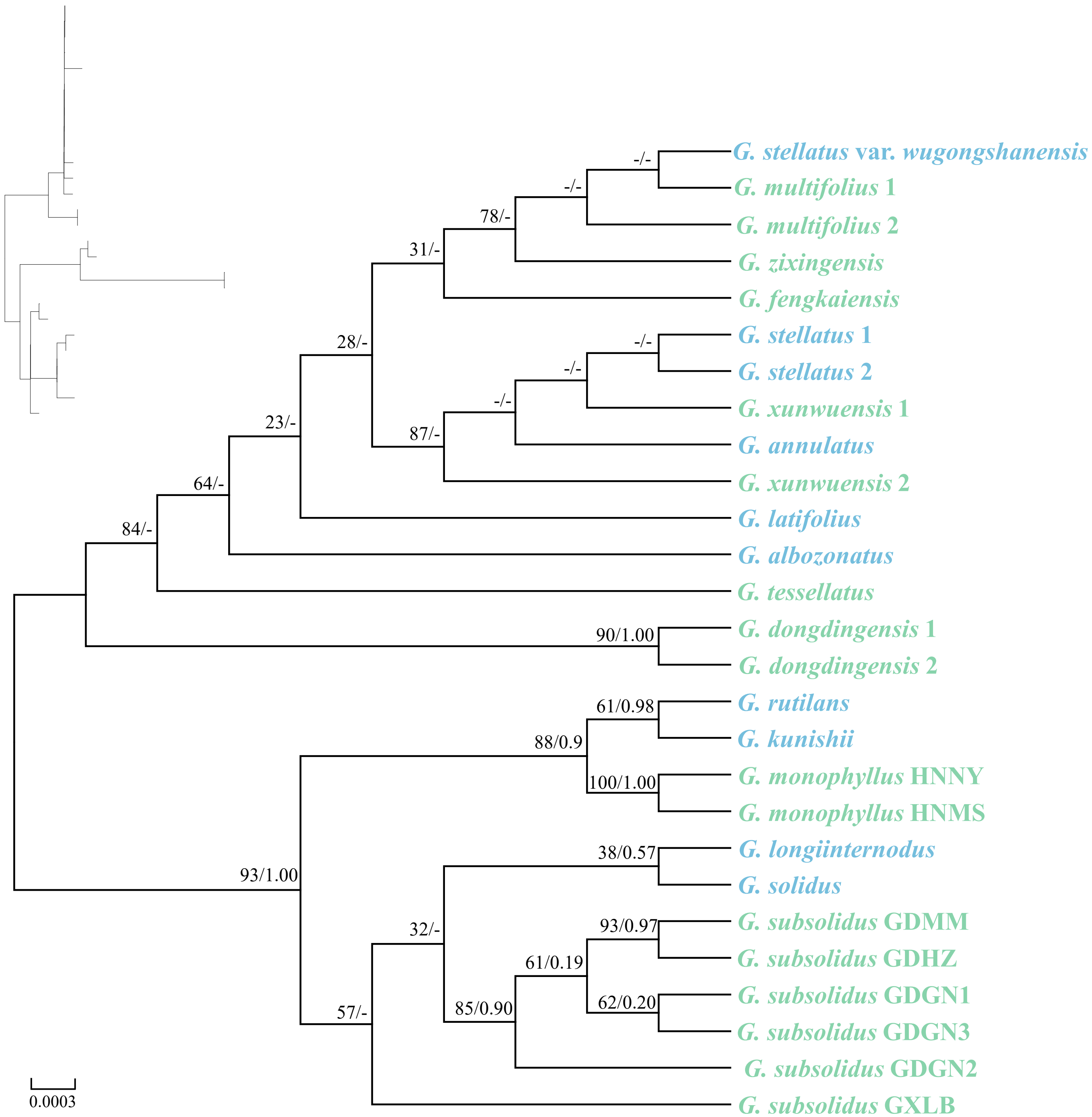

Supplementary Figure 4 Phylogenetic tree of 27 taxa using maximum likelihood (ML) and Bayesian inference (BI) methods based on nrDNA repeats.

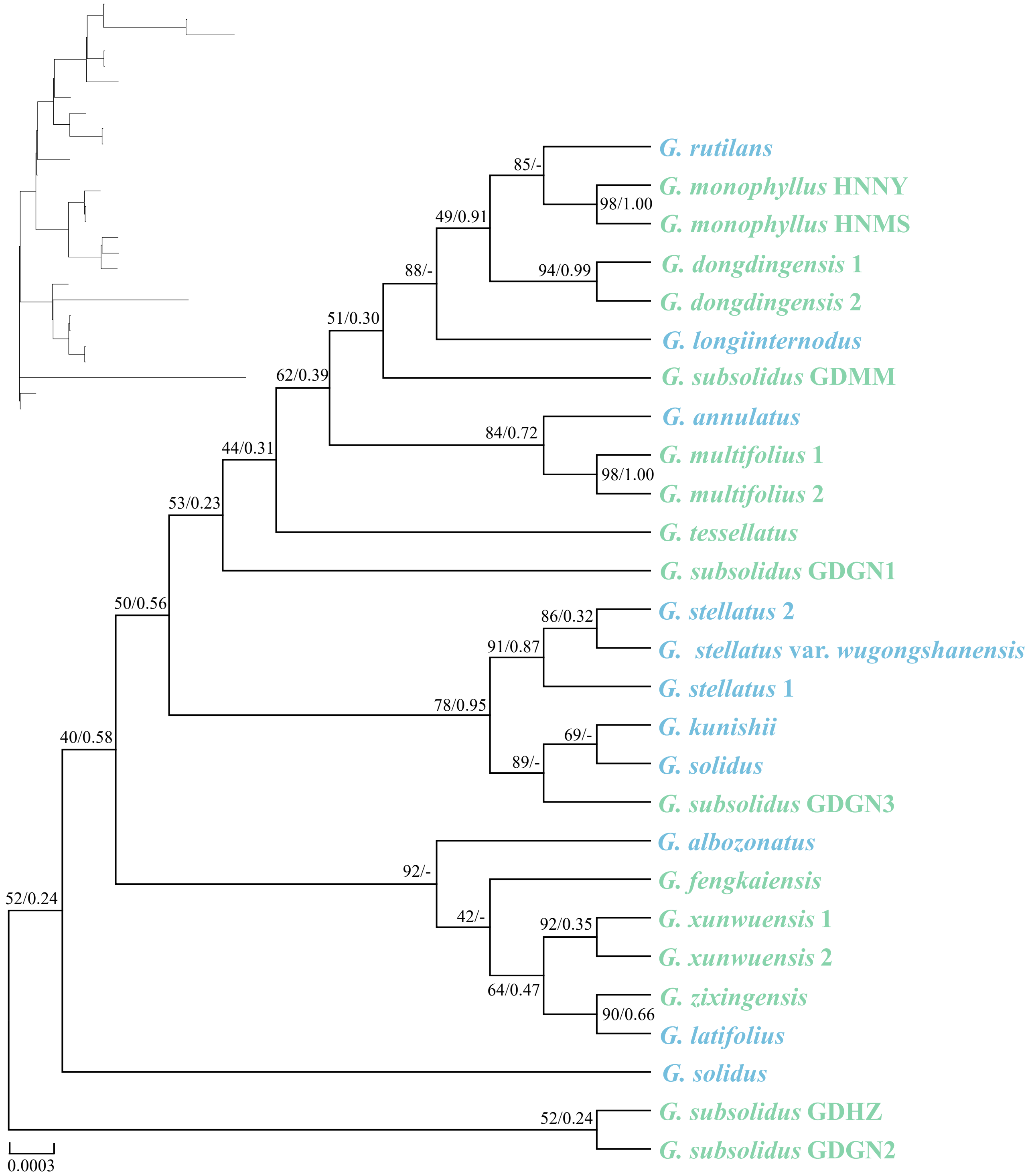

Supplementary Figure 5 Phylogenetic tree of 27 taxa using maximum likelihood (ML) and Bayesian inference (BI) methods based on 24-shared mitochondrial protein-coding genes.

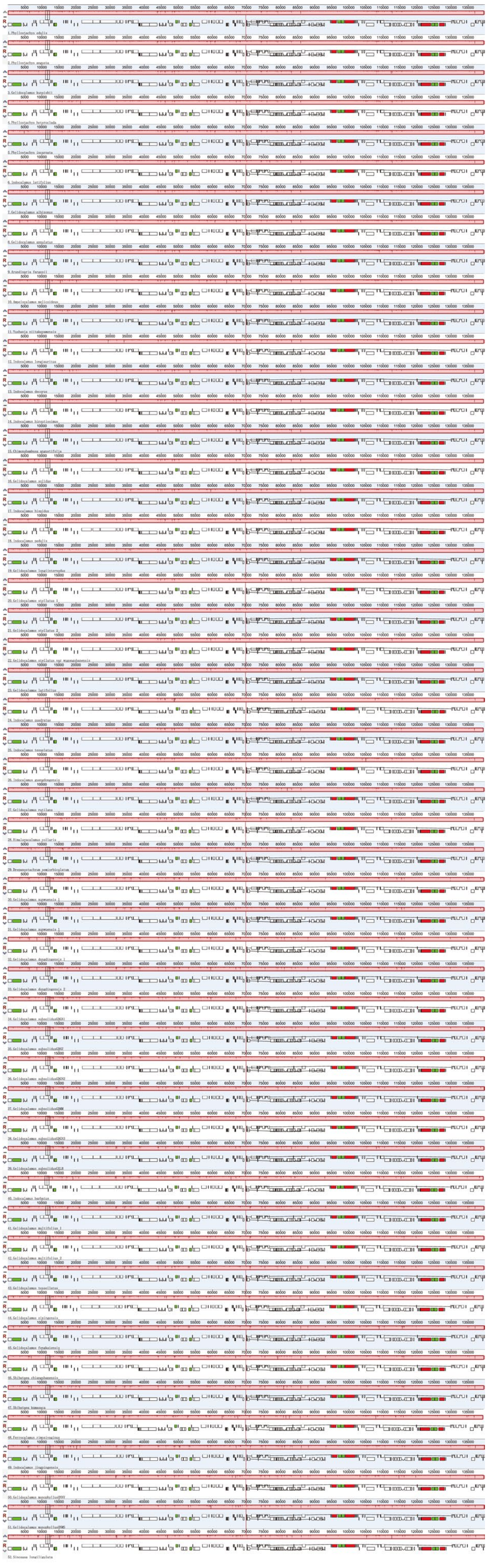

Supplementary Figure 6 The colinearity between the chloroplast genomes of the two clades.

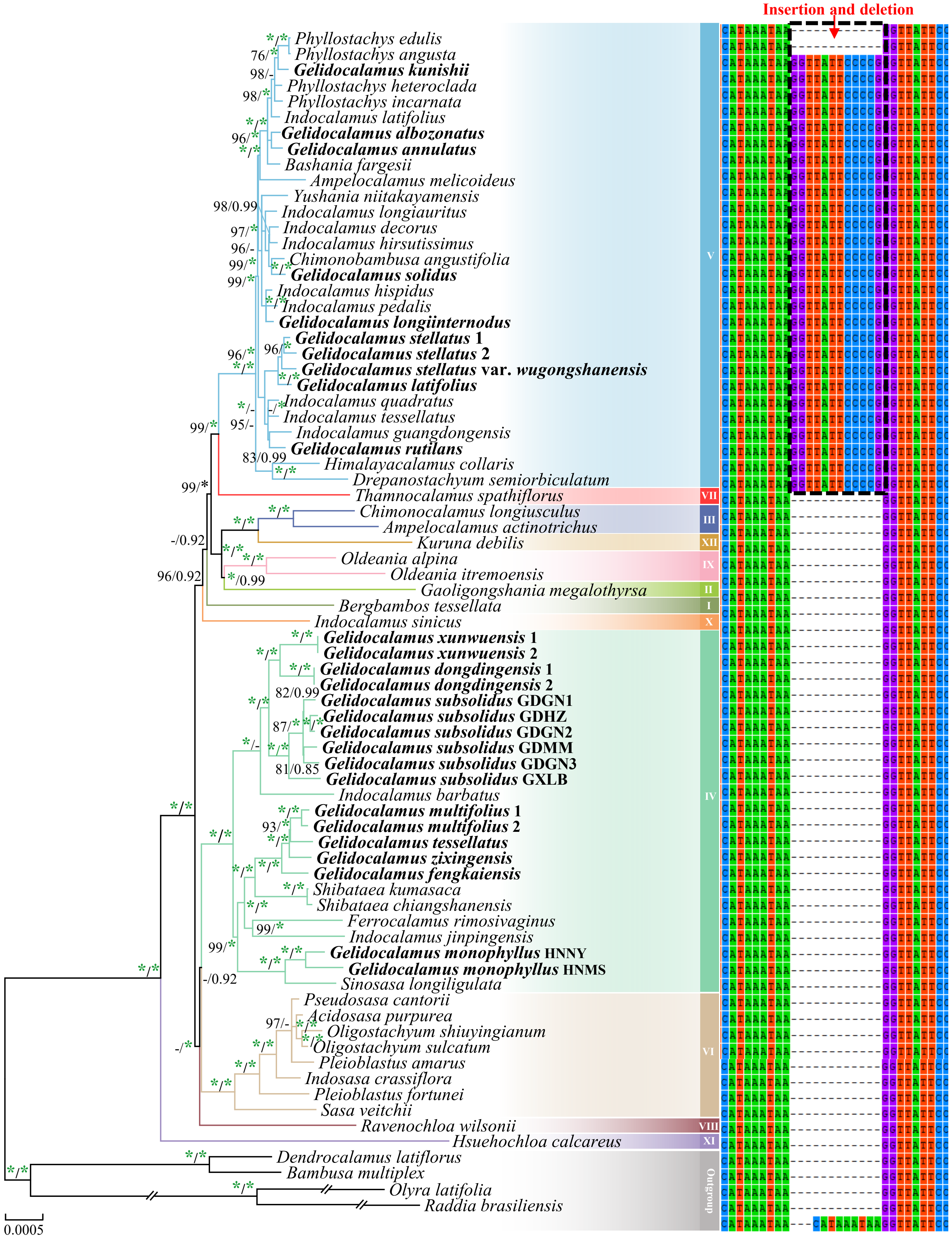

Supplementary Figure 7 The insertion sequence "GGTTATTCCCCG" located at the LSC/IRb junction.

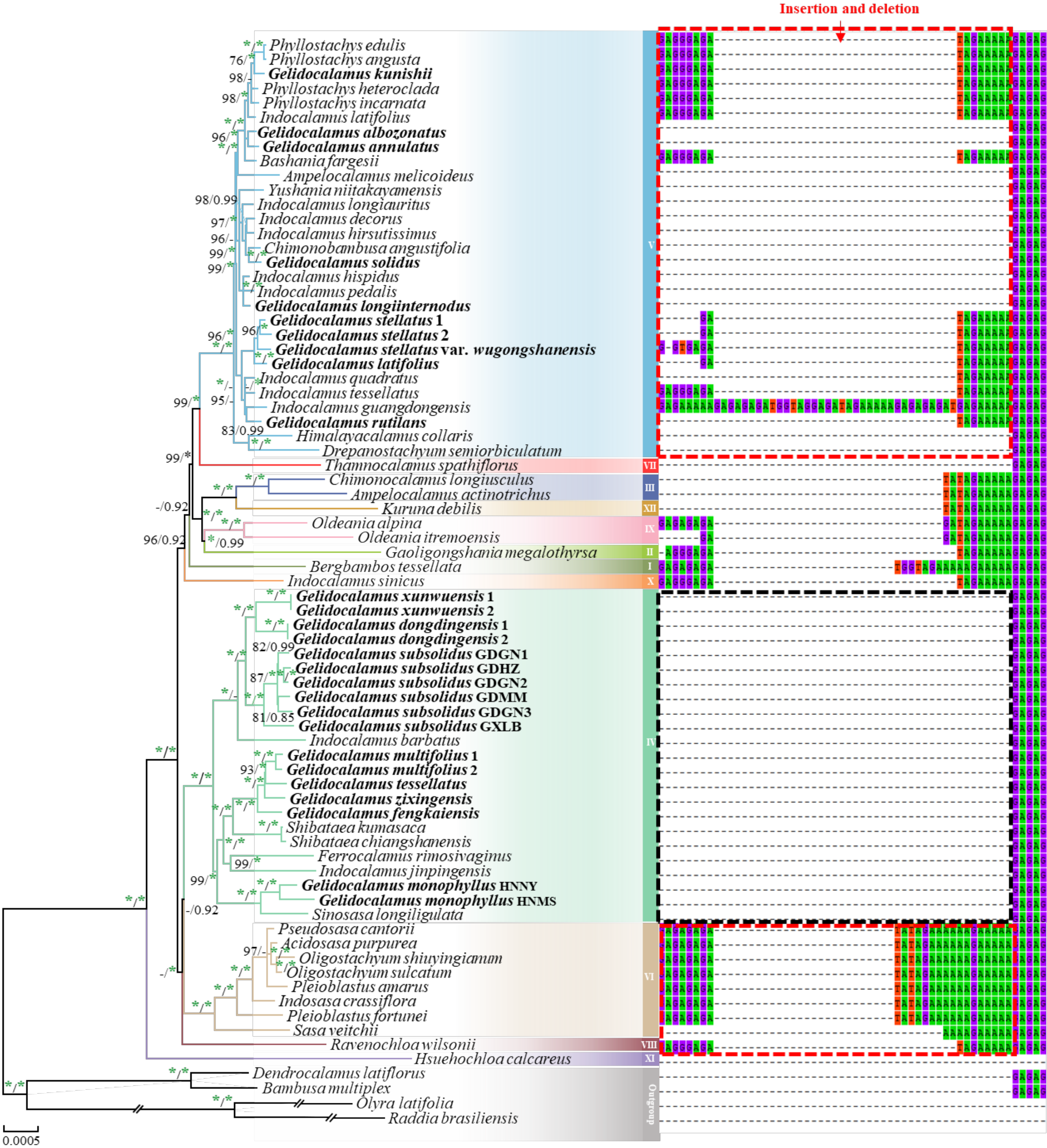

Supplementary Figure 8 The insertion sequence "GAGGGGAGATAGAAAAA" located at the LSC/IRb junction.

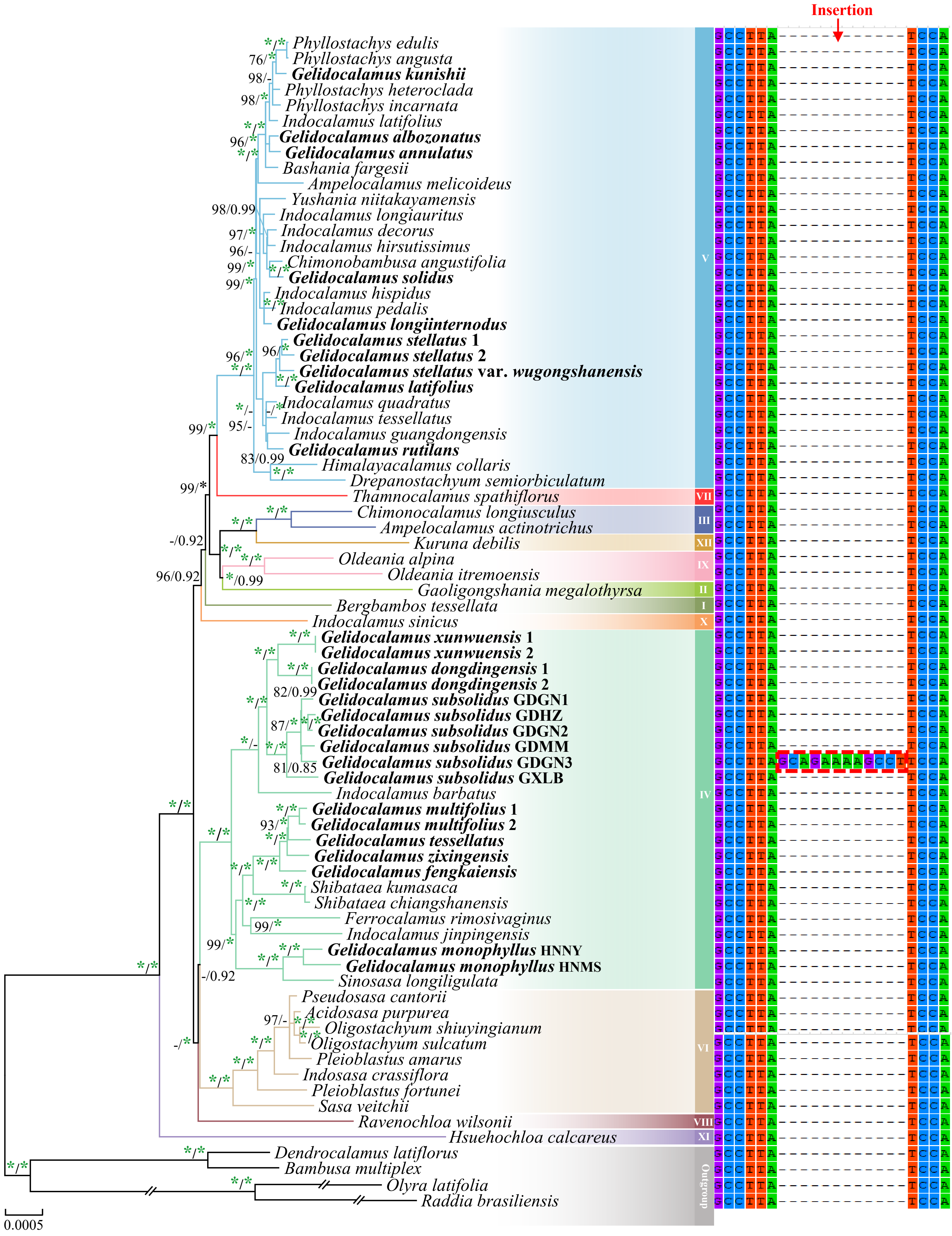

Supplementary Figure 9 The insertion sequence "GCAGAAAAGCCT" located at the LSC/IRb junction.

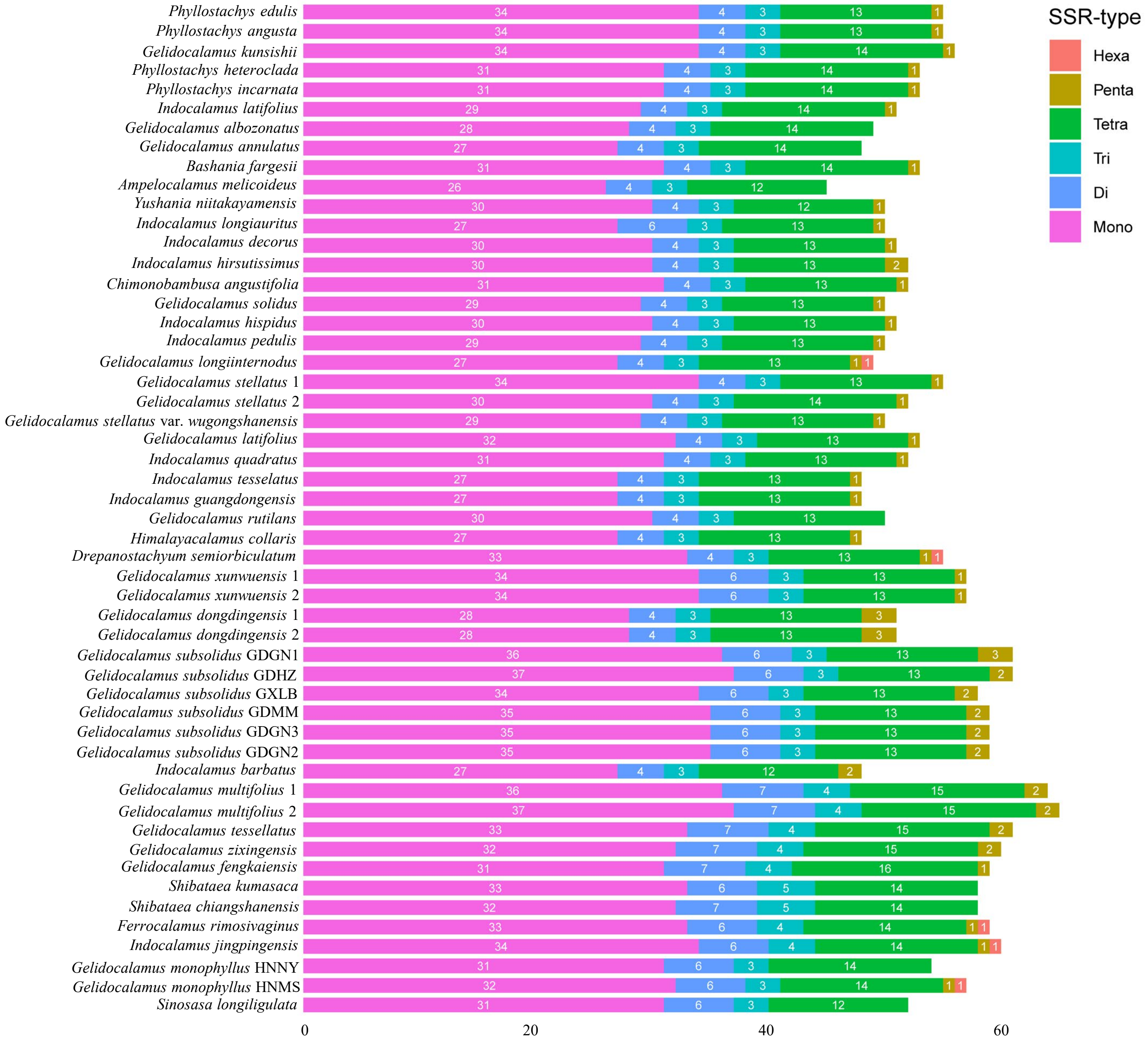

Supplementary Figure 10 Number of six types of SSRs repeat.

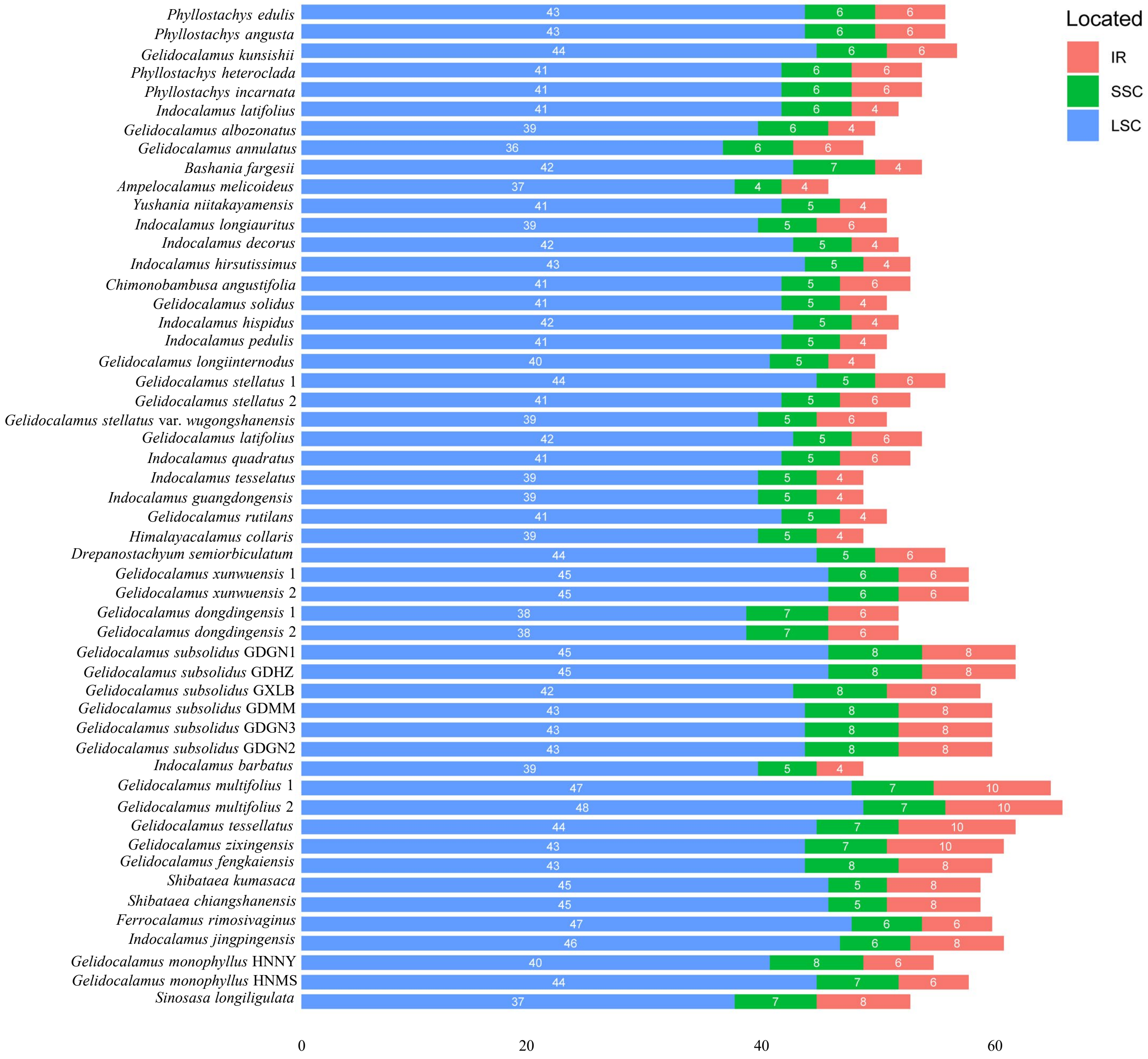

Supplementary Figure 11 Number of SSRs in LSC, SSC, and IR regions.

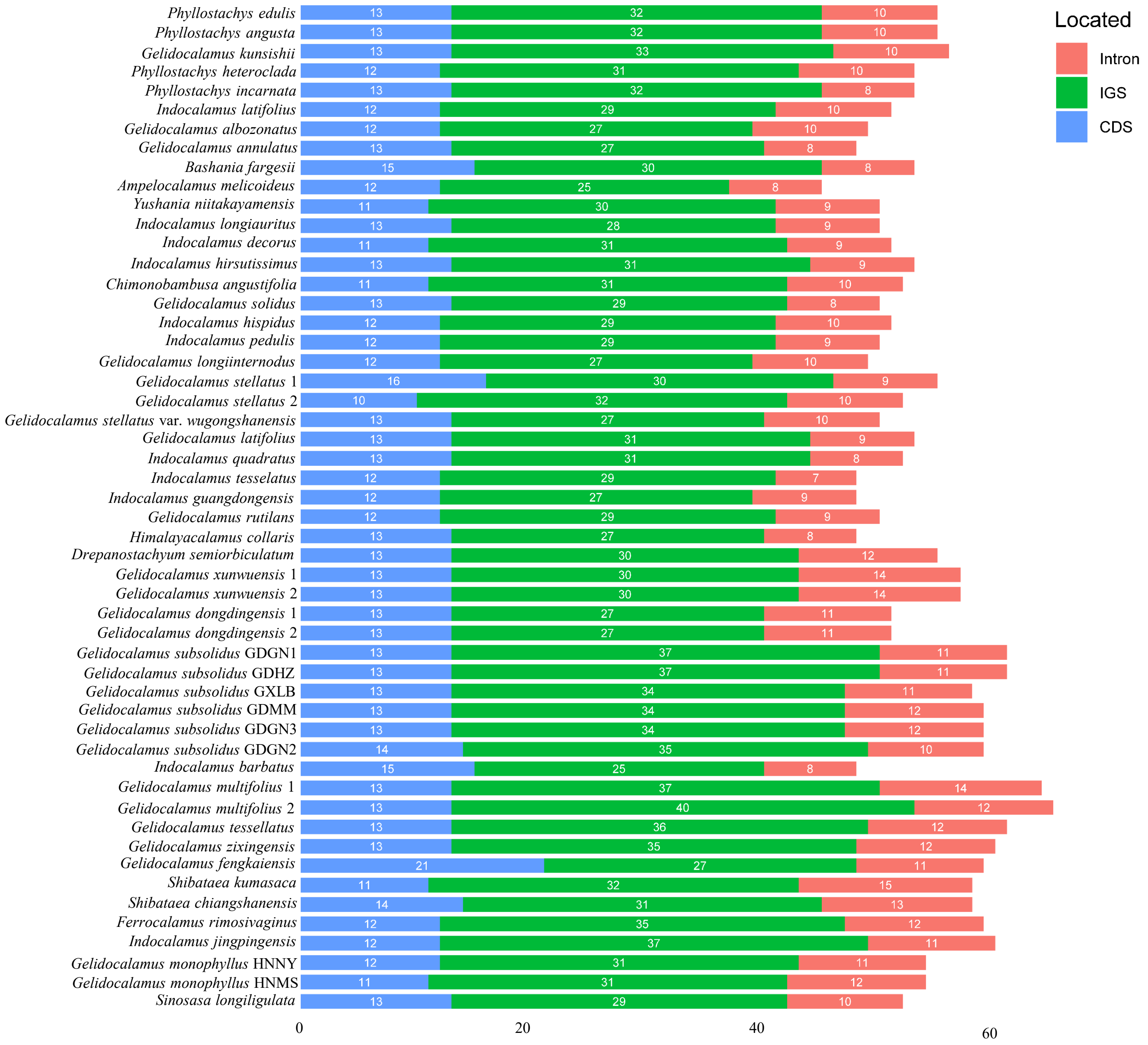

Supplementary Figure 12 Number of SSRs in the coding regions (CDS), intergenic region (IGS), and introns.

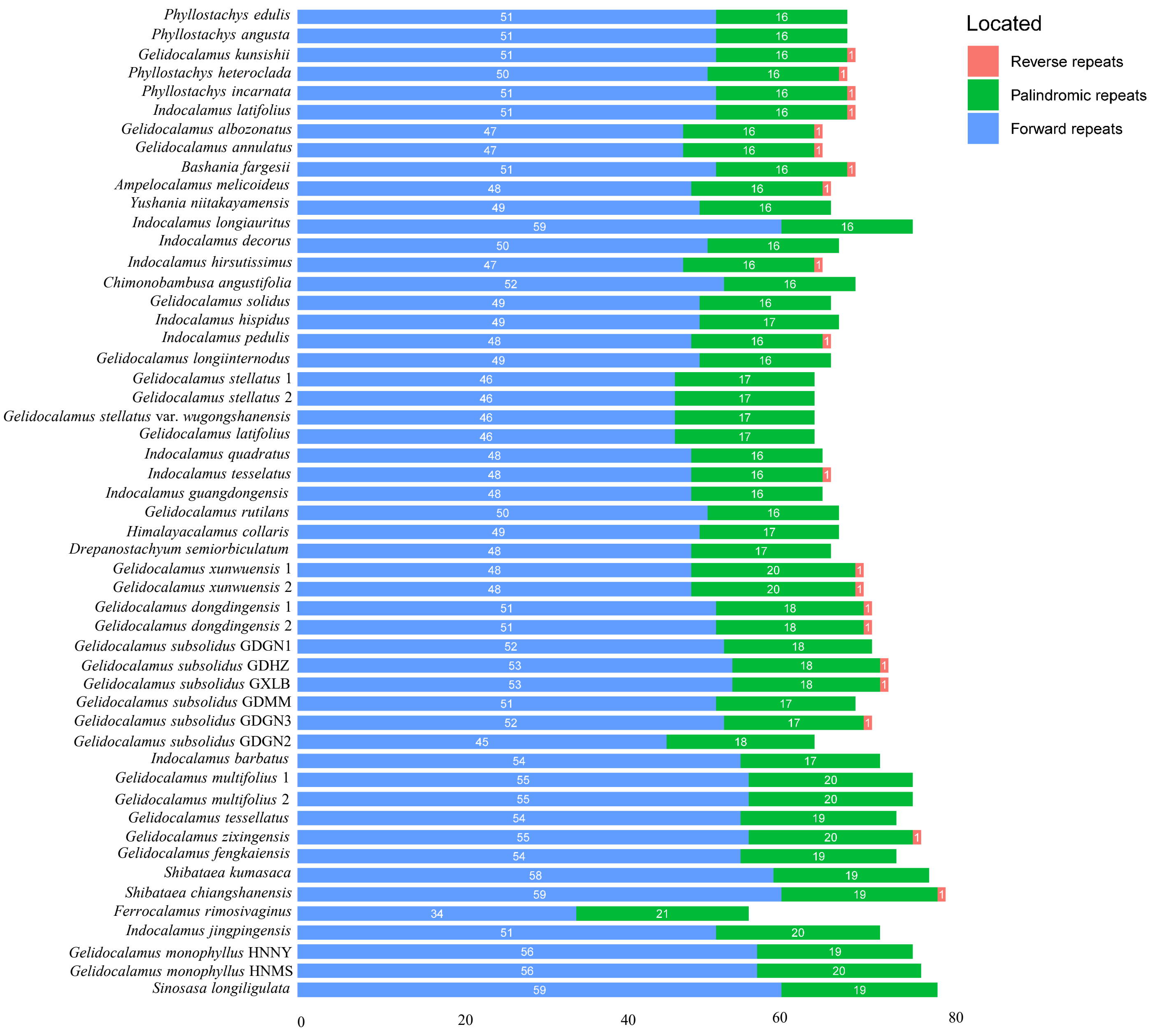

Supplementary Figure 13 Number of forward, palindromic, and reverse repeats.

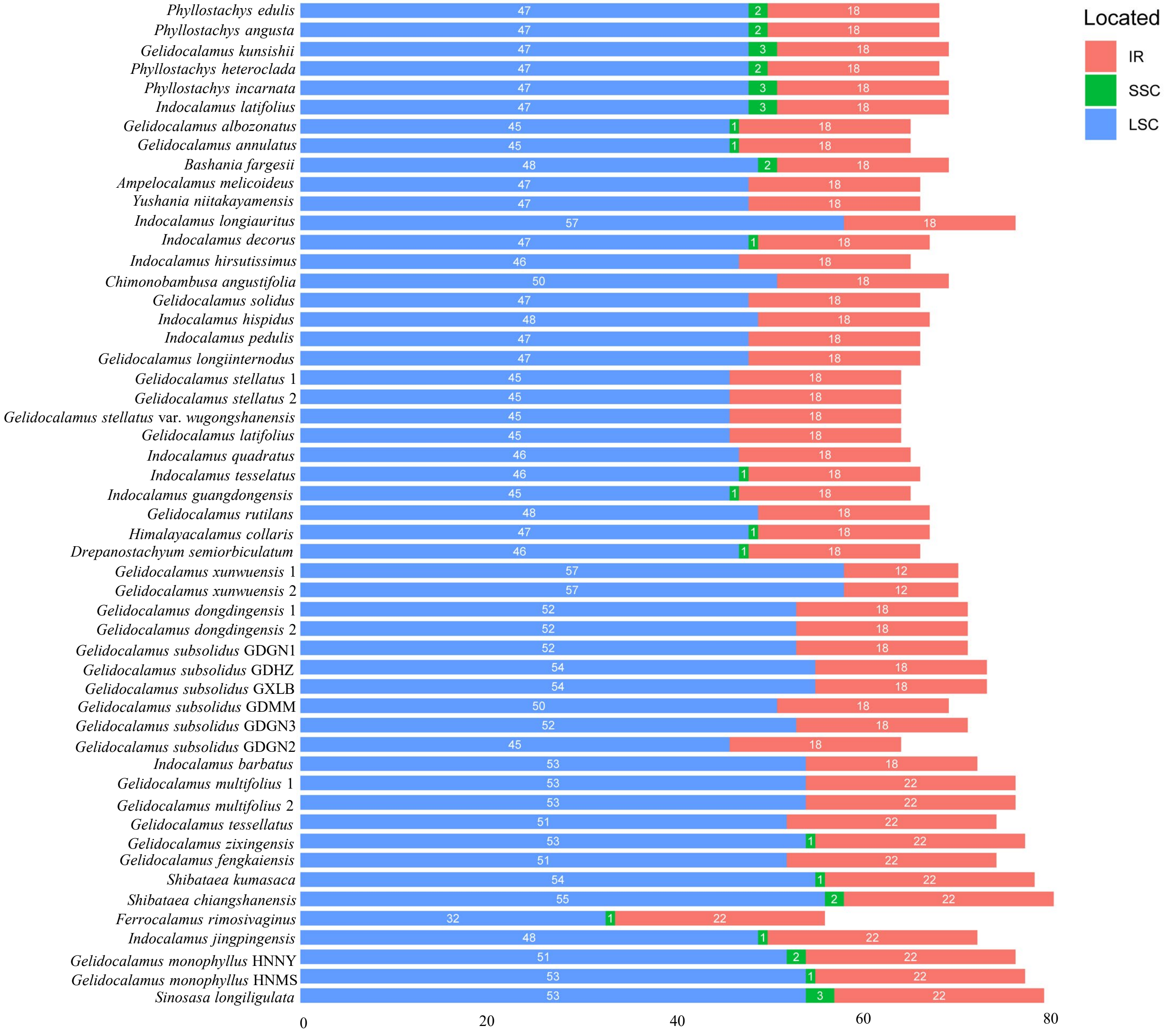

Supplementary Figure 14 Number of long repeats in in LSC, SSC, and IR regions.

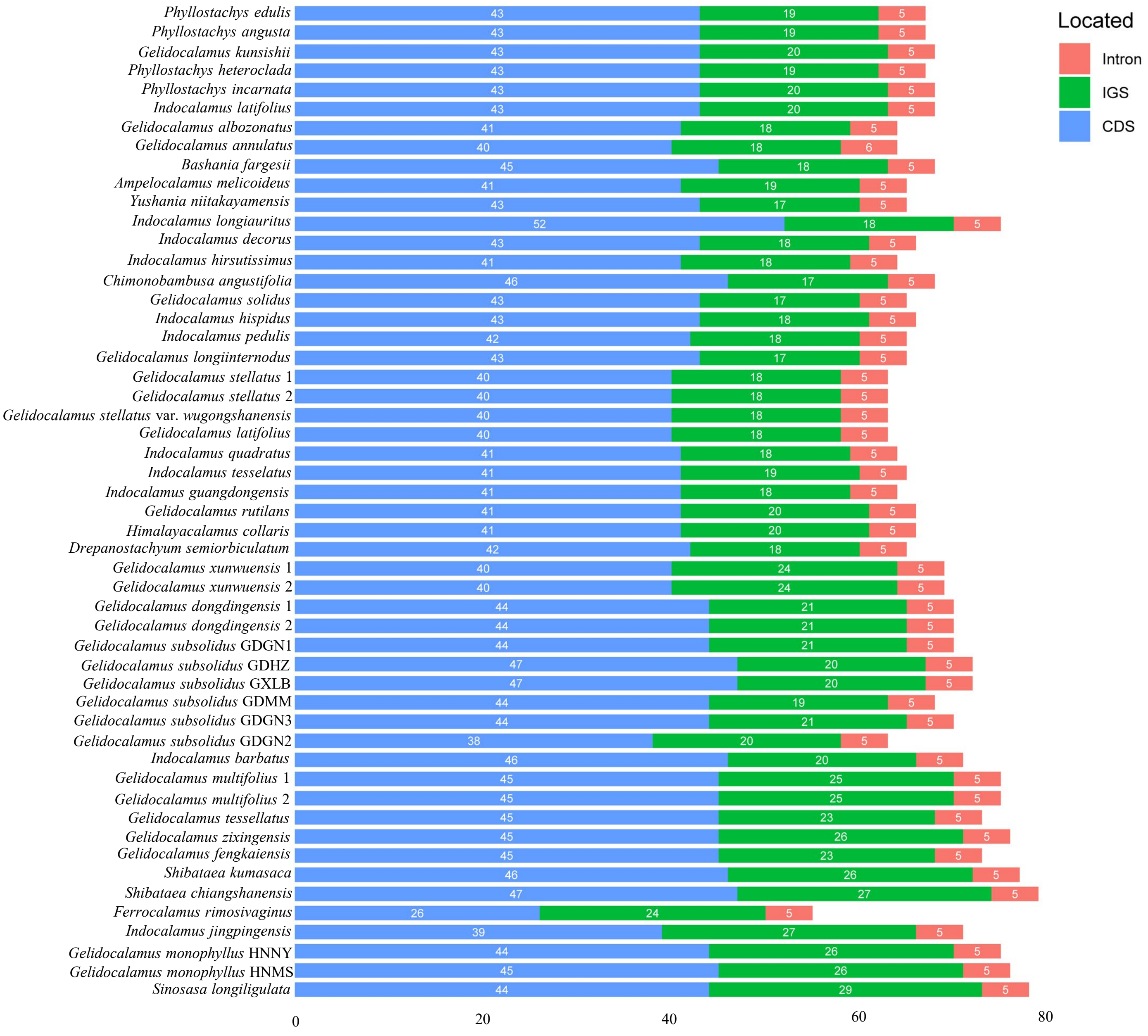

Supplementary Figure 15 Number of long repeats in the coding regions (CDS), intergenic region (IGS), and introns.
